# Phosphatidylthreonine and Lipid-Mediated Control of Parasite Virulence

**DOI:** 10.1371/journal.pbio.1002288

**Published:** 2015-11-13

**Authors:** Ruben D. Arroyo-Olarte, Jos F. Brouwers, Arunakar Kuchipudi, J. Bernd Helms, Aindrila Biswas, Ildiko R. Dunay, Richard Lucius, Nishith Gupta

**Affiliations:** 1 Department of Molecular Parasitology, Humboldt University, Berlin, Germany; 2 Department of Biochemistry and Cell Biology, Institute of Biomembranes, Utrecht University, The Netherlands; 3 Institute of Medical Microbiology, Otto von Guericke University, Magdeburg, Germany; 4 Parasitology Unit, Max-Planck Institute for Infection Biology, Berlin, Germany; University of Vermont, UNITED STATES

## Abstract

The major membrane phospholipid classes, described thus far, include phosphatidylcholine (PtdCho), phosphatidylethanolamine (PtdEtn), phosphatidylserine (PtdSer), and phosphatidylinositol (PtdIns). Here, we demonstrate the natural occurrence and genetic origin of an exclusive and rather abundant lipid, phosphatidylthreonine (PtdThr), in a common eukaryotic model parasite, *Toxoplasma gondii*. The parasite expresses a novel enzyme PtdThr synthase (*Tg*PTS) to produce this lipid in its endoplasmic reticulum. Genetic disruption of *Tg*PTS abrogates de novo synthesis of PtdThr and impairs the lytic cycle and virulence of *T*. *gondii*. The observed phenotype is caused by a reduced gliding motility, which blights the parasite egress and ensuing host cell invasion. Notably, the PTS mutant can prevent acute as well as yet-incurable chronic toxoplasmosis in a mouse model, which endorses its potential clinical utility as a metabolically attenuated vaccine. Together, the work also illustrates the functional speciation of two evolutionarily related membrane phospholipids, i.e., PtdThr and PtdSer.

## Abstract 2

An exclusive membrane lipid, phosphatidylthreonine, is revealed to be naturally abundant in the widespread protist parasite *Toxoplasma gondii*, where it has evolved adaptively and is essential for parasite virulence.

## Author Summary

Lipids are essential constituents of biological membranes, and most organisms across the tree of life use a relatively limited repertoire of lipids in their membranes. This work reveals the natural and abundant presence of an exclusive lipid phosphatidylthreonine (PtdThr) in *Toxoplasma gondii*, a ubiquitous protozoan parasite of humans and animals. PtdThr is made by a novel parasite-specific enzyme, PtdThr synthase, which has evolved from the widespread enzyme phosphatidylserine synthase. The study shows that PtdThr is required for asexual reproduction and virulence of the parasite in vivo, and a metabolically attenuated mutant strain of *Toxoplasma* lacking PtdThr can protect vaccinated mice against acute and currently incurable chronic infection. This discovery demonstrates adaptive “speciation” of PtdThr from an otherwise near-universal membrane lipid phosphatidylserine and reveals de novo PtdThr synthesis in *T*. *gondii* as a potential drug target.

## Introduction

Intracellular protozoan parasites impose a substantial threat to human and animal health. *Toxoplasma gondii* is one of the most prevalent protozoan parasites, infecting nearly all warm-blooded vertebrates, including humans [1]. Over the last two decades, *T*. *gondii* has also become a popular model organism to understand the biology of parasitic and free-living protozoans alike. The parasite causes debilitating opportunistic infections in immunocompromised individuals and neonates. The disease occurs by the multiplication and persistence of its acute and chronic stages, the latter of which is impervious to host immunity and existing drugs. Acute infection, hallmarked by tissue necrosis, is caused by successive rounds of lytic cycles, comprising host cell invasion, intracellular replication, and egression [1]. The entry and exit of *T*. *gondii* into and from host cells is dependent on calcium-regulated gliding motility and exocytosis of specialized secretory organelles [2,3].

Parasites proliferating within their host cells oblige a substantial biogenesis of organelle membranes, which are composed of mainly phospholipids and neutral lipids. The typical and natural phospholipids characterized so far include phosphatidylcholine (PtdCho), phosphatidylethanolamine (PtdEtn), phosphatidylserine (PtdSer), phosphatidylinositol (PtdIns), phosphatidylglycerol, and phosphatidate [4]. Others and we have shown that *T*. *gondii* contains common eukaryotic phospholipids as well as the pathways for autonomous synthesis [5–8]. Physiological functions of phospholipids in the parasite are poorly understood however, and most of the underlying enzymes have not been characterized as yet. Moreover, despite a steadily rising interest in roles of lipids in host–pathogen interactions [9], the existence and biogenesis of divergent pathogen-specific lipids remain very much underappreciated.

## Results

### *T*. *gondii* Contains an Exclusive As Well As Major Phospholipid, Phosphatidylthreonine

**Fig 1:**
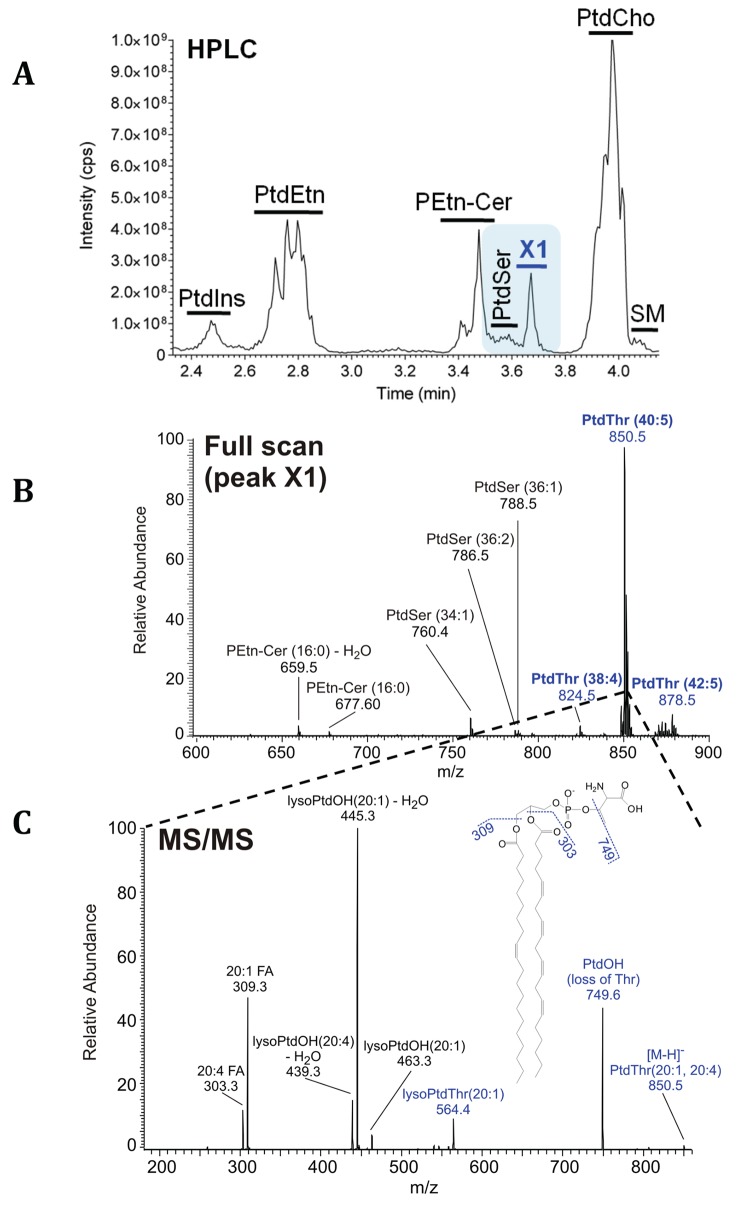
Lipidomics of *T*. *gondii* tachyzoites identifies a novel parasite lipid, PtdThr. **(A)** Elution profile showing the retention times and relative abundance of lipids isolated from extracellular tachyzoites (10^7^). X1 represents a previously unknown lipid. **(B)** MS analysis of X1 fraction revealing PtdThr, PtdSer, and PEtn-Cer species. Individual lipids were identified by their fragmentation patterns and *m/z* ratios in the negative ionization mode. **(C)** MS/MS spectrum of X1-derived major peak (*m/z* 850.5) from panel B. Note the neutral loss of 101 Da (transition from 850.5 to 749.6). Acyl chains (*sn*-1, 20:1; *sn*-2, 20:4) were identified by their masses.

In our expedition to characterize membrane biogenesis in *T*. *gondii*, we fractionated the parasite lipids by high-performance liquid chromatography (HPLC) and observed a major lipid peak X1 eluting next to PtdSer (Fig 1A). Other major lipids were PtdCho, PtdEtn, PtdIns, PtdSer, and phosphoethanolamine-ceramide (PEtn-Cer), confirming previous reports [5,7]. To determine the precise identity of X1 fraction, we executed mass spectrometry (MS) analysis, which revealed certain PEtn-Cer and PtdSer species, as expected (Fig 1B). The most prominent peak in this fraction with an *m/z* of 850.5, however, did not correspond to a PEtn-Cer or PtdSer species. Tandem MS of the indicated peak showed a neutral loss of 101 atomic mass units (*m/z*, 749.6) contrary to the expected 87 for serine, or 141 for ethanolamine (Fig 1C). The *m/z* profile matched to threonine as the polar head group instead, which was also independently confirmed by HPLC analysis of amino acid derived from lipid hydrolysis (S1A Fig). The fatty acyl chains of this particular lipid, phosphatidylthreonine (PtdThr henceforth), were identified as 20:1 and 20:4. Other detectable, but evidently minor, PtdThr species also contained comparably polyunsaturated and long acyl chains (Fig 1B).

Next, we resolved the parasite lipids by two-dimensional thin layer chromatography (TLC). As apparent (S1B Fig), and also shown elsewhere [5], PtdCho, PtdEtn, PtdIns and PtdSer (besides PtdThr) were the major parasite lipids visualized by iodine-vapor staining. PtdThr (X1), detected again near PtdSer, was authenticated by MS analysis (S1C Fig). PtdThr accounted for ≈20 nmol/10^8^ parasites by lipid phosphorus quantification. It is noteworthy that PtdThr has been previously reported as a rare and notably minor PtdSer analog in certain mammalian cells and selected prokaryotes [10–13]. It was also shown that the base-exchange-type PtdSer synthase activity located in the ER and its mitochondria-associated membranes in mammalian cells normally uses serine as its primary substrate [14,15]; but it can produce PtdThr as a by-product under serine-deprived condition [10]. In contrast, our results reveal a surprisingly abundant and natural occurrence of PtdThr in a widespread protist.

### A Novel PtdThr Synthase Localized Likely in the Endoplasmic Reticulum of *T*. *gondii* Synthesizes PtdThr

PtdThr species were absent in uninfected human fibroblasts used to culture parasites (S2 Fig), which implied their de novo synthesis in *T*. *gondii*. Our in silico and PCR (polymerase chain reaction) analyses aimed at establishing the genetic origin of PtdThr identified two putative base-exchange-type PtdSer synthases in the parasite database (www.ToxoDB.org; TGGT1_273540, TGGT1_261480) encoding for 614 and 540 residues, which we designated as *Tg*PTS (PtdThr synthase) and *Tg*PSS (PtdSer synthase), respectively, based on the results described in this work. Unlike PSS occurring across the phyla, orthologs of PTS could only be found in selected parasitic (*Neospora*, *Eimeria*, *Phytophtora*) and free-living (*Perkinsus*) chromalveolates (S3 Fig). Of note is the fact that distinct asparagine, histidine, and cysteine residues are conserved in all PSS orthologs, but not in *Tg*PTS, which contains substitutions to glutamate, tryptophan, and serine at the equivalent positions (S4 Fig). Phylogeny supported the variability in the substrate-binding pocket of PSS [16] with that of PTS sequences and indicated a loss of latter enzyme in other related parasites.

**Fig 2:**
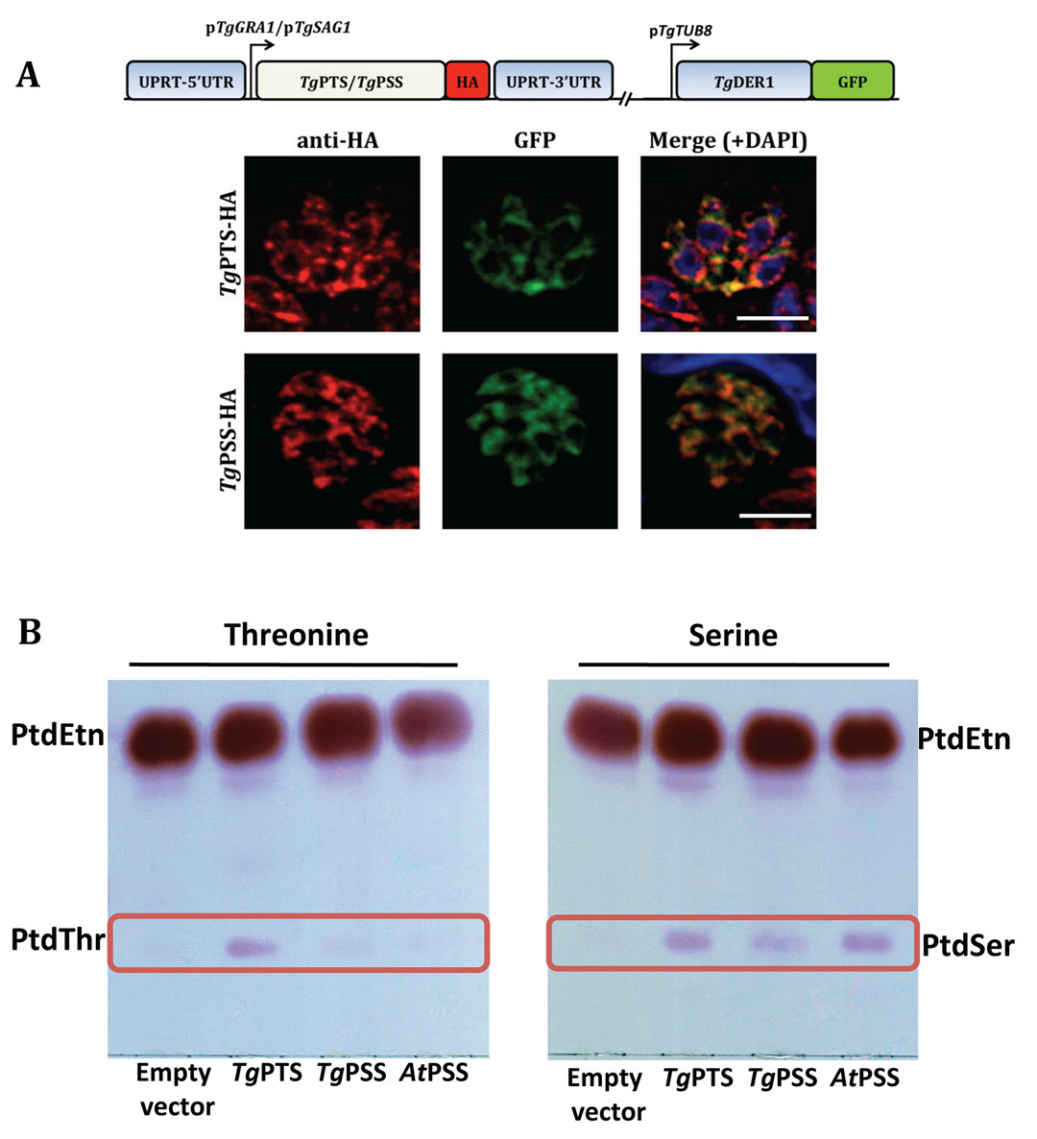
PtdThr and PtdSer are synthesized by PTS and PSS in the ER of *T*. *gondii*. **(A)** Immunostained images of the HA-tagged PtdThr synthase (*Tg*PTS) and PtdSer synthase (*Tg*PSS) targeted at the uracil phosphoribosyltransferase (UPRT) locus and expressed under the control of the regulatory elements of *TgGRA1* or *TgSAG1*, respectively. Colocalization was done with *Tg*Der1-GFP (ER marker). Yellow fluorescence in the merged panel indicates expression of *Tg*PTS-HA and *Tg*PSS-HA in the ER (bars, 5 μm). No crossfluorescence from green to red channel or vice versa was observed. For costaining with other organelle markers, refer to S5 and S11 Figs. **(B)** TLC-resolved lipid profiles of *E*. *coli* strains harboring the specified expression constructs. To assess the *Tg*PTS and *Tg*PSS activities, ORFs (open reading frames) were cloned into the M15/pREP4 strain of *E*. *coli* and expression was induced by IPTG (isopropyl β-D-1-thiogalactopyranoside) in cultures supplemented with 5 mM threonine or serine. Total lipids were resolved in chloroform/methanol/acetate (130:50:20) and visualized by ninhydrin spray.

Ectopic expression of epitope-tagged *Tg*PTS-HA and *Tg*PSS-HA showed a marked distribution in the endoplasmic reticulum (ER) of the parasite (Fig 2A). Because overexpression under the control of a foreign promoter may cause localization artifacts, we detected endogenous levels of PSS and PTS in transgenic parasite lines, in which the corresponding genes had been tagged with HA-epitope at the 3’-ends. As discussed below (S10B and S11 Figs), PSS fusion protein regulated by its promoter localized mainly in the parasite ER/mitochondrion intersecting with each other, and to some extent in acidocalcisomes/plant-like vacuole. The native expression of PTS was too low to be visualized (not shown). We nonetheless tested potential localization of PTS in other organelles using the parasites overexpressing *Tg*PTS-HA; however, we found no apparent signal in micronemes, rhoptries, dense granules, mitochondrion, apicoplast, and acidocalcisomes/plant-like vacuole (S5 Fig). To evaluate the enzymatic function of both enzymes, we expressed them in *Eschericia coli* and assessed their catalytic activity in the presence of serine or threonine (Fig 2B). Lipid analyses of bacterial strains harboring empty vector (negative control), *Tg*PTS, *Tg*PSS, or *Arabidopsis thaliana* PSS (positive control [17]) showed synthesis of PtdSer by *At*PSS and *Tg*PSS as well as by *Tg*PTS when using serine as substrate. Unlike *At*PSS and *Tg*PSS, however, *Tg*PTS also produced PtdThr in presence of threonine, indicating that *Tg*PSS is indeed a PtdSer synthase, whereas *Tg*PTS can synthesize both PtdThr and PtdSer.

### The *Δtgpts* Mutant Lacks Autonomous Synthesis of PtdThr

**Fig 3:**
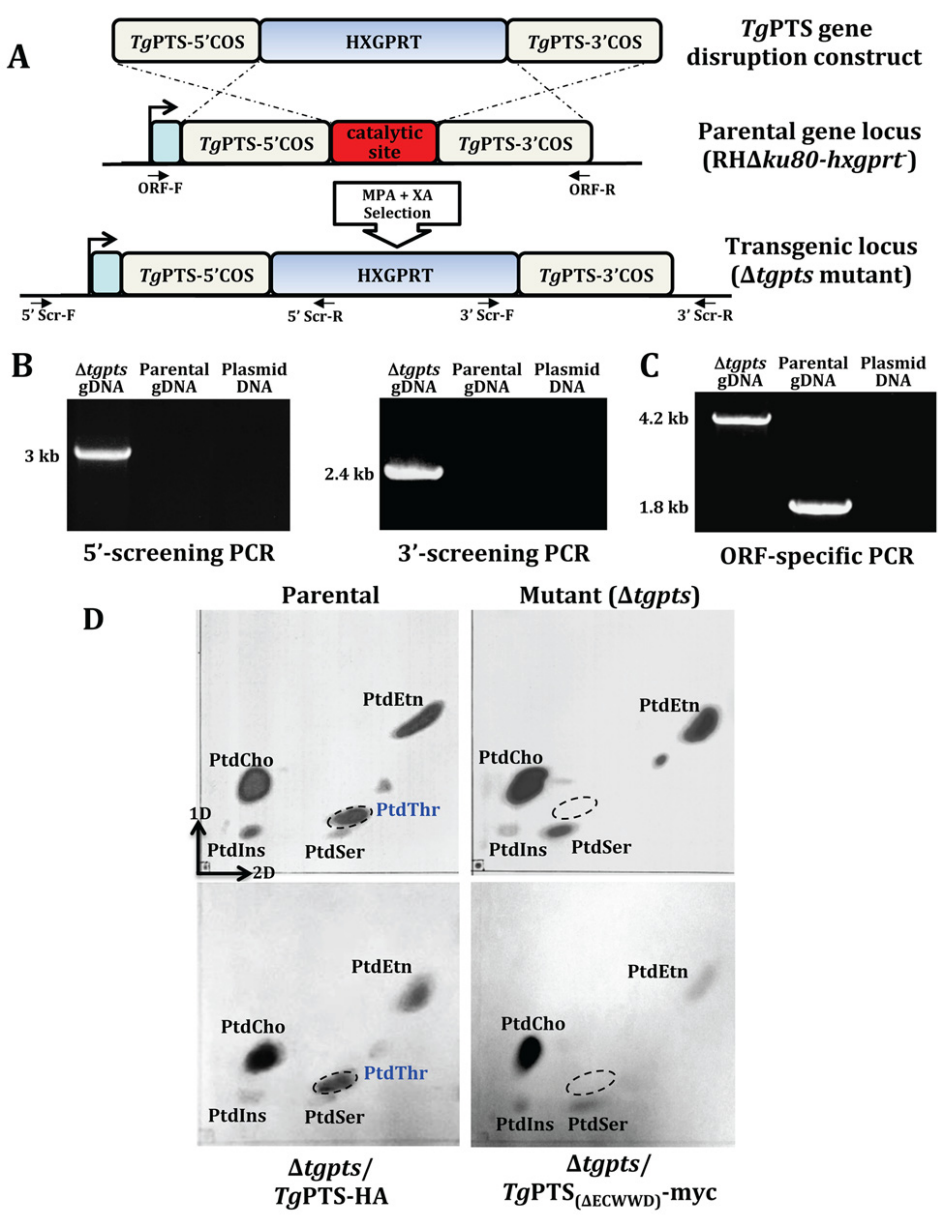
The *Δtgpts* strain is devoid of de novo PtdThr synthesis. **(A)** Scheme for the targeted disruption of the *TgPTS* gene by double homologous recombination. The plasmid contained 5’ and 3’ crossover sequences (COS) flanking the hypoxanthine-xanthine-guanine phosphoribosyltransferase (HXGPRT) marker, which allows resistance to mycophenolic acid (MPA) and xanthine (XA). The PTS-disrupted (Δ*tgpts*) strain lacking the conserved ECWWD (Glu-Cys-Trp-Trp-Asp) residues was identified by 5’ and 3’ screening primers (5’Scr-F/R, 3’Scr-F/R). **(B)** PCR images of a typical Δ*tgpts* mutant showing specific amplification of the DNA bands by 5’ (3 kb) and 3’ (2.4 kb) genomic screening. The parental gDNA and plasmid were included as negative controls. **(C)** ORF-specific PCR confirming a successful insertion of the selection marker at the *TgPTS* gene locus. PCR shows amplification of an expected 4.2 kb band in the Δ*tgpts* strain as opposed to the expected 1.8 kb in the parental parasites, and none in the plasmid DNA (negative control). Identity of all PCR amplicons was confirmed by sequencing. **(D)** Lipid profiles of the indicated strains by two-dimensional TLC. Total lipids (0.8–1 x 10^8^ parasites) were resolved and detected by iodine-vapor staining. PtdThr band (encircled) is absent in the Δ*tgpts* strain. It is restored by a wild-type *Tg*PTS (*Tg*PTS-HA), but not by a catalytically-inactive ΔECWWD isoform (*Tg*PTS_(ΔECWWD)_-myc). Note that it appears as though PtdSer is not increased too much in the *Tg*PTS_(ΔECWWD)_-myc-complemented mutant when compared to the parental strain. A sustained culture of the Δ*tgpts*/*Tg*PTS_(ΔECWWD)_-myc strain has proven particularly difficult due to severe growth defect (S9 Fig), likely caused by a dominant-negative effect exerted on PSS in an already attenuated mutant.

To endorse the function of *Tg*PTS in *T*. *gondii*, we disrupted the gene in the parasite genome (Fig 3A). The Δ*tgpts* strain was isolated by recombination-specific PCR screening, which confirmed an efficient disruption of the PTS gene locus (Fig 3B). Accordingly, the ORF-specific primers amplified a band of 4.2 kb in the Δ*tgpts* strain in lieu of the expected 1.8 kb in the parental parasites (Fig 3C), which corroborated the targeted insertion of the selection marker and deletion of the predicted catalytic site (342ECWWD346; S4 Fig) [16]. Moreover, expression of adjacent transcripts flanking the *PTS* gene was unaffected, further confirming the specificity of transgenic manipulation (S6 Fig).

**Fig 4:**
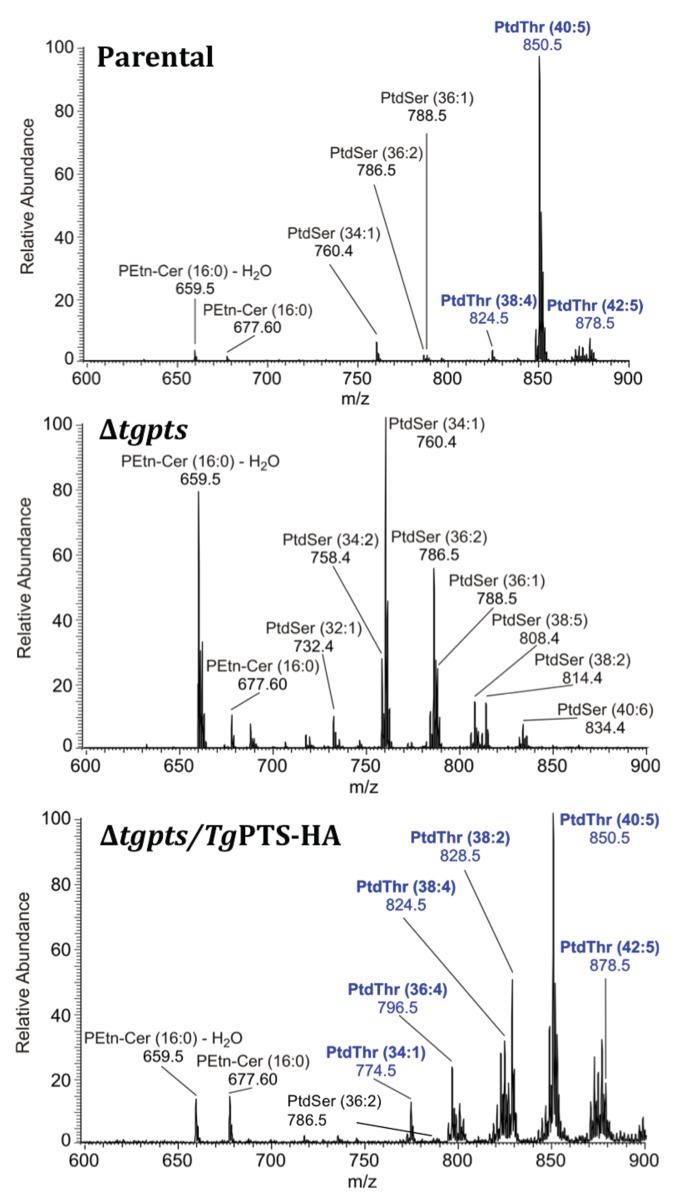
The *Δtgpts* strain lacks all detectable PtdThr species and shows an evident increase in PtdSer. Total lipids (10^7^ tachyzoites) of the specified parasite strains were resolved by HPLC (not shown), and the eluted X1 fraction was subjected to MS analysis, as described in Fig 1. Exemplified spectra of the parental (top), Δ*tgpts* (middle), and complemented (bottom) strains confirm the absence of a major (*m/z* 850.5, 40:5) and two minor (*m/z* 824.5, 38:4; *m/z*, 878.5, 42:5) PtdThr species in the Δ*tgpts* strain. PtdSer-derived peaks are more intense in the Δ*tgpts* strain, which is consistent with TLC (Fig 3D) and lipid phosphorus assays (S7 Fig). Unlike the parental strain, the Δ*tgpts* mutant overexpressing *Tg*PTS-HA lacks certain PtdSer species and shows additional minor PtdThr species, which is likely due to mutual regulation of PSS and PTS catalysis.

Synthesis of PtdThr was abrogated in the Δ*tgpts* strain, as shown by TLC and lipid phosphorus assays (Fig 3D, S7 Fig). Concurrently, we observed a 3-fold gain in PtdSer level that was proportionate to the content of PtdThr in the parental strain (S7 Fig). This observed increase in PtdSer amount was due to an increased de novo synthesis of lipid, as shown by metabolic labeling with radioactive serine (S8 Fig). All these effects were entirely reversible as complementation of the mutant with a functional *Tg*PTS recovered PtdThr (Fig 3D), as well as restored a normal PtdSer synthesis and lipid content (S7 and S8 Figs). Consistent with these results, the MS analyses revealed the absence of all PtdThr species and a parallel increase in PtdSer species in the mutant, which were reverted in the PTS-complemented strain (Fig 4). Taken together, these data show an autonomous synthesis of PtdThr in *T*. *gondii* and its abolition in the Δ*tgpts* mutant. They also indicate a mutual regulation of PSS and PTS enzymes.

### Disruption of the *TgPTS* Gene Impairs the Lytic Cycle of *T*. *gondii*

**Fig 5:**
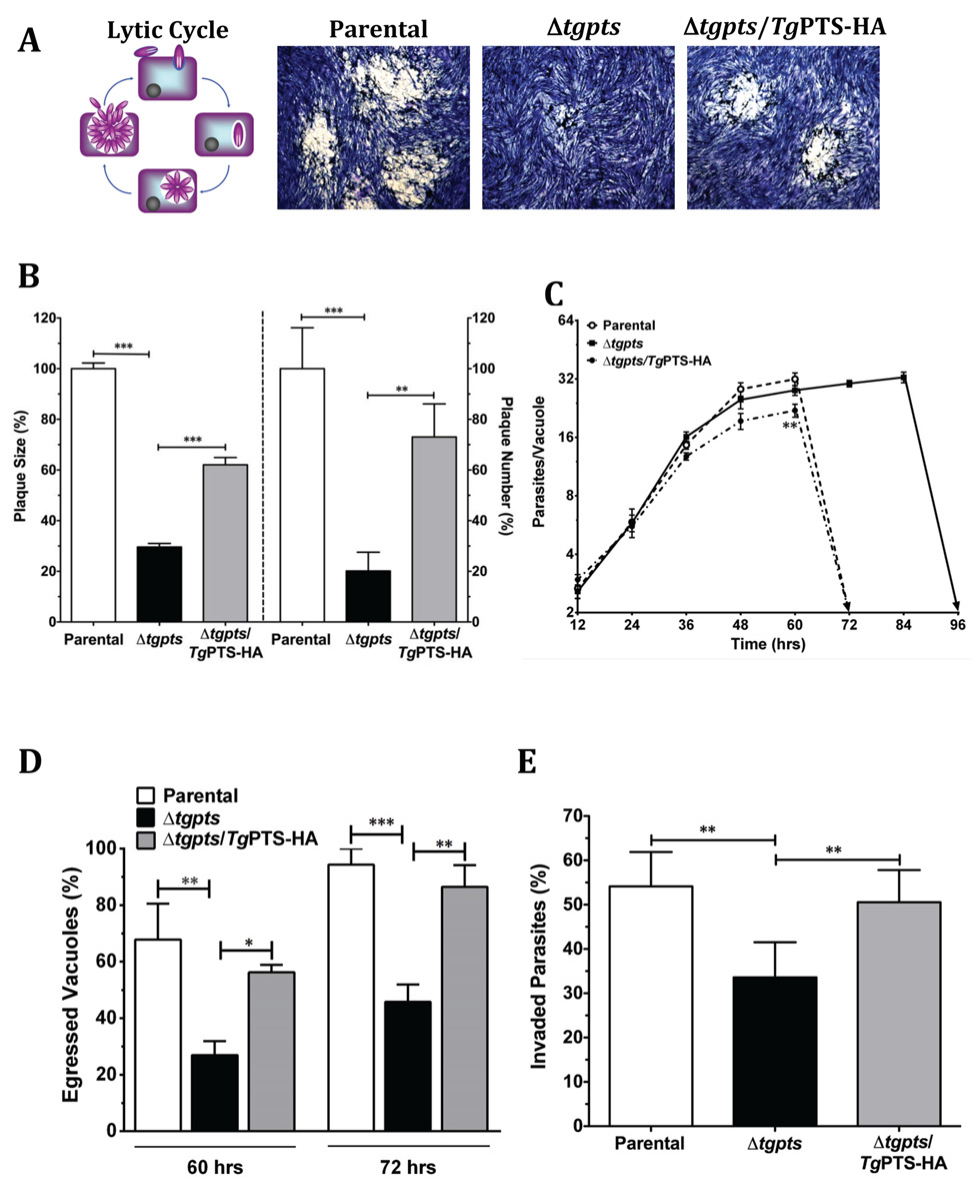
The *Δtgpts* mutant is defective in egress and invasion but not in replication. **(A)** Representative images showing the in vitro growth fitness of the parental, Δ*tgpts*, and PTS-complemented strains by plaque assays, which recapitulate successive lytic cycles of tachyzoites in host cells (see schematics). The mutant was generated as shown in Fig 3. Complemented strain expressed wild-type *Tg*PTS-HA under the control of the *TgGRA1* promoter at the *TgUPRT* gene locus. **(B)** Quantification of plaque area (*left Y-axis*) and numbers (*right Y-axis*). 120–300 plaques of each strain from 7 assays were scored. **(C)** Intracellular replication of the specified strains, as deduced by the mean number of parasites/vacuoles at different periods. A total of 100–200 vacuoles were analyzed for each strain (*n* = 4 assays). **(D)** The natural egress of the indicated parasite strains at 60 and 72 hr postinfection (MOI, 1). In total, 200–300 vacuoles were counted for each strain (*n* = 4 assays). **(E)** Invasion rates of the parasite strains (700–1,000 parasites of each strain from 5 assays). The number of egressed vacuoles and invaded parasites were estimated by dual-color staining, as described in Materials and Methods. Graphs in panels B–E indicate the mean ± standard error of the mean (SEM) (*p < 0.05, **p < 0.01, ***p < 0.001). Note that a partial rescue of plaque growth (panel B) in the complemented strain as opposed to near complete recovery of invasion and egress defects in panels D–E is caused by a mild replication defect due to overexpression of PTS in the Δ*tgpts* mutant (panel C).

We next assessed the physiological impact of *Tg*PTS ablation on the parasite growth by plaque assays. Compared to the parental strain, the Δ*tgpts* strain formed noticeably smaller (−70%) and considerably fewer (−80%) plaques (Fig 5A and 5B). Ectopic expression of wild-type *Tg*PTS largely rescued the parasite growth. In contrast, the catalytically-dead isoform of *Tg*PTS_(ΔECWWD)_, which was incapable of restoring PtdThr level in the Δ*tgpts* strain (Fig 3D), could not amend the growth defect (S9 Fig), confirming the physiological need of the PTS activity for the parasite. It should be mentioned that the Δ*tgpts* strain expressing *Tg*PTS_(ΔECWWD)_-myc showed an accentuated growth defect when compared to the mutant (S9A Fig), which prevented its prolonged culture and detailed biochemical analyses.

**Fig 6:**
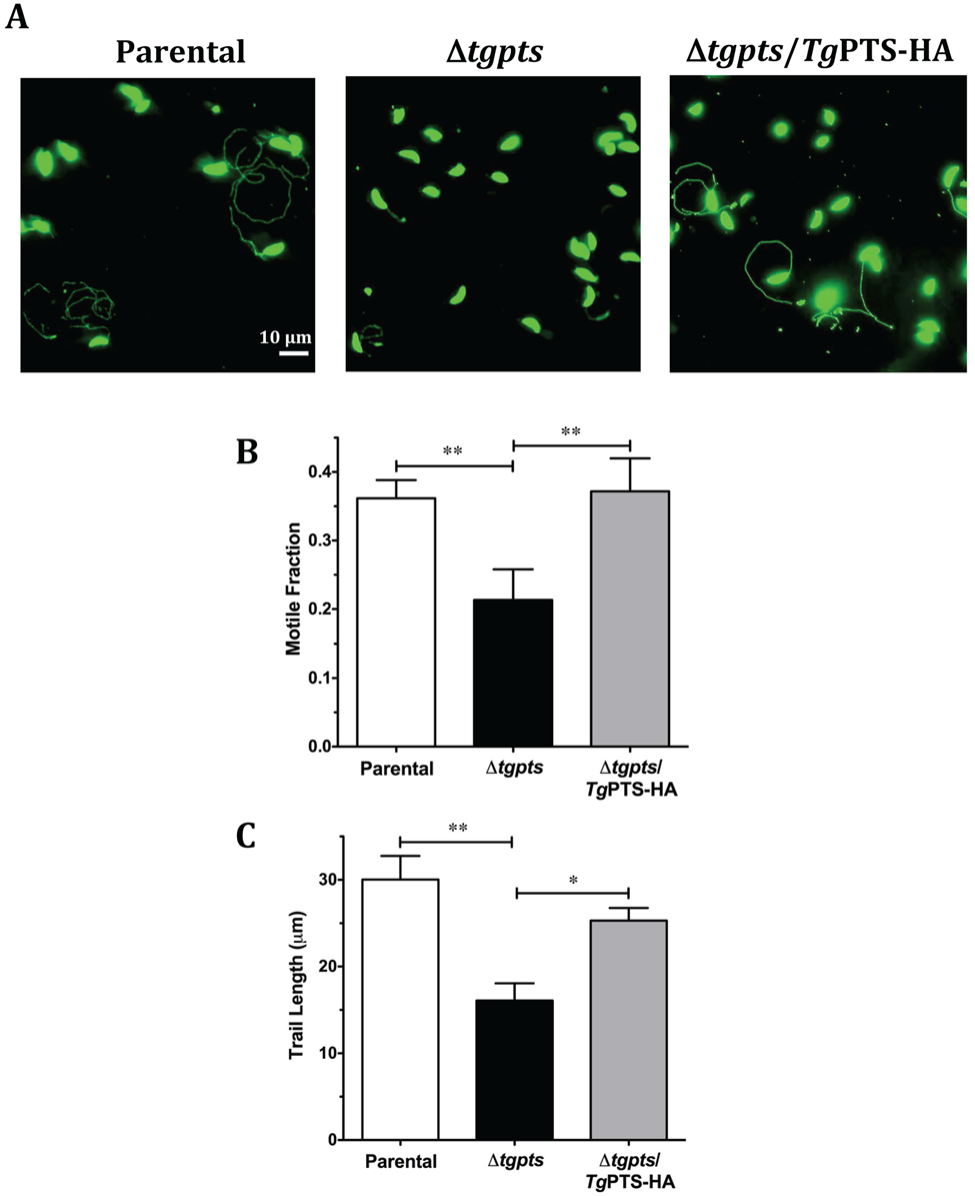
The *Δtgpts* mutant displays an attenuated gliding motility. **(A)** Images showing the motility of the parental, Δ*tgpts*, and PTS-complemented strains. Fresh extracellular tachyzoites were allowed to glide on glass coverslips (15 min, 37°C) and then stained with anti-*Tg*Sag1 and Alexa488 antibodies. **(B–C)** Motile fractions and trail lengths of the indicated strains, as deduced from *Tg*Sag1 staining of parasites in panel A. In total, 700–1,000 parasites of each strain were scored from 4–6 experiments (mean ± SEM; *p < 0.05, **p < 0.01).

In-depth phenotyping of the parental, Δ*tgpts* mutant, and PTS-complemented parasite strains revealed a normal replication in the mutant (Fig 5C). Surprisingly, however, a complete lysis of host cells by the mutant was markedly delayed up to 96 hr, as opposed to 72 hr in host fibroblasts infected with the parental and complemented strains (dotted and solid arrows, Fig 5C). In accord, the mutant displayed a much slower natural egress than the two control strains (Fig 5D). For example, only about 27% and 46% of the mutant vacuoles were disrupted after 60 and 72 hr of infection as opposed to 67% and 94% of the parental vacuoles. Noticeably, the mutant was also impaired in invading fresh host cells (Fig 5E). Egression and invasion events require gliding motility in *T*. *gondii*, which drives the parasite’s exit from dilapidated host cells and ensuing infection of neighboring cells [2]. Indeed, the Δ*tgpts* strain displayed an evidently reduced motility, as determined by a lower motile fraction and shorter trails compared to the two reference strains (Fig 6). These assays demonstrate the mandatory requirement of PTS activity for an effective functioning of the lytic cycle in *T*. *gondii*.

### Growth Impairment in the *Δtgpts* Mutant Is Not due to an Increased Content of PtdSer

**Fig 7:**
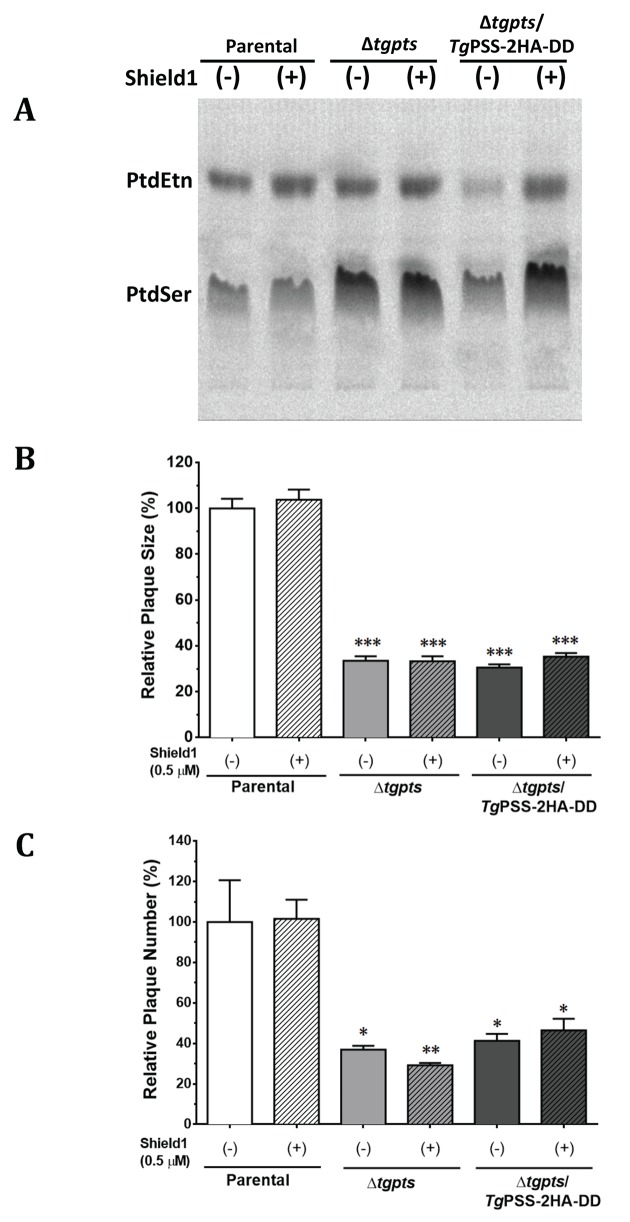
Knockdown of PSS in the *Δtgpts*/*Tg*PSS-2HA-DD strain does not rescue growth defect caused by the loss of PTS alone. **(A)** Autoradiography of TLC-resolved lipids after labeling of the indicated strains with radioactive serine. Parasites were cultured during the intracellular phase without or with Shield1 (0.5 μM, 24 hr) followed by labeling of extracellular parasites (5 x 10^7^) with ^14^C-serine (2 μCi, 100 μM, 2 hr, 37°C). Lipids were resolved in chloroform/ethanol/water/triethylamine (30:35:7:35). For corresponding quantitative radiolabeling and phospholipid analysis, refer to S12 Fig. **(B–C)** Relative growth fitness of the parasite strains incubated with or without Shield1. Plaque assays were executed and quantified, as described in Materials and Methods. Note that growth of the Δ*tgpts/TgPSS-2HA-DD* and Δ*tgpts* strains are equivalent irrespective of Shield1 in cultures. Statistics was performed with respect to the untreated parental strain (mean ± SEM, *n* = 3 assays; *p < 0.05, **p < 0.01, ***p < 0.001).

To examine whether an elevated level of PtdSer underlies the observed growth phenotype in the PTS mutant, we created a double mutant (Δ*tgpts*/*Tg*PSS-2HA-DD; S10A Fig). The *TgPSS* gene was fused with a Shield1-regulated degradation domain (DD) and 2HA epitopes at 3’-end [18] to achieve a conditional expression of PSS protein. The PSS-2HA-DD fusion protein showed a predominant fluorescent signal in the ER (S10B Fig). We also observed apparent staining of PSS with the markers of mitochondrion (F1B) [19] and acidocalcisomes/plant-like vacuole (vacuolar proton pyrophosphatase 1; VP1) [20], whereas other organelles, micronemes, rhoptries, dense granules, and apicoplast did not show evident PSS staining (S11 Fig). Expression of PSS-2HA-DD could be regulated by exposure to Shield1 (S10B and S10C Fig). Metabolic labeling of parasite lipids with serine (PtdSer and nascent decarboxylated product PtdEtn) also confirmed that PSS activity was restored to the parental level in the absence of Shield1 (Fig 7A, S12A Fig). A knockdown of PSS activity reinstated PtdSer content in the Δ*tgpts* strain (S12B Fig). Even a normal PtdSer pool, however, was unable to rectify the growth defect in the Δ*tgpts*/*Tg*PSS-2HA-DD double mutant, which mirrored plaques formed by the Δ*tgpts* strain (Fig 7B and 7C). These results exclude the impact of amplified PtdSer in disrupting the lytic cycle, while strengthening the physiological importance of PtdThr for *T*. *gondii*.

### The *Δtgpts* Strain Is Defective in Virulence and Protects Mice against Acute and Chronic Toxoplasmosis

**Fig 8:**
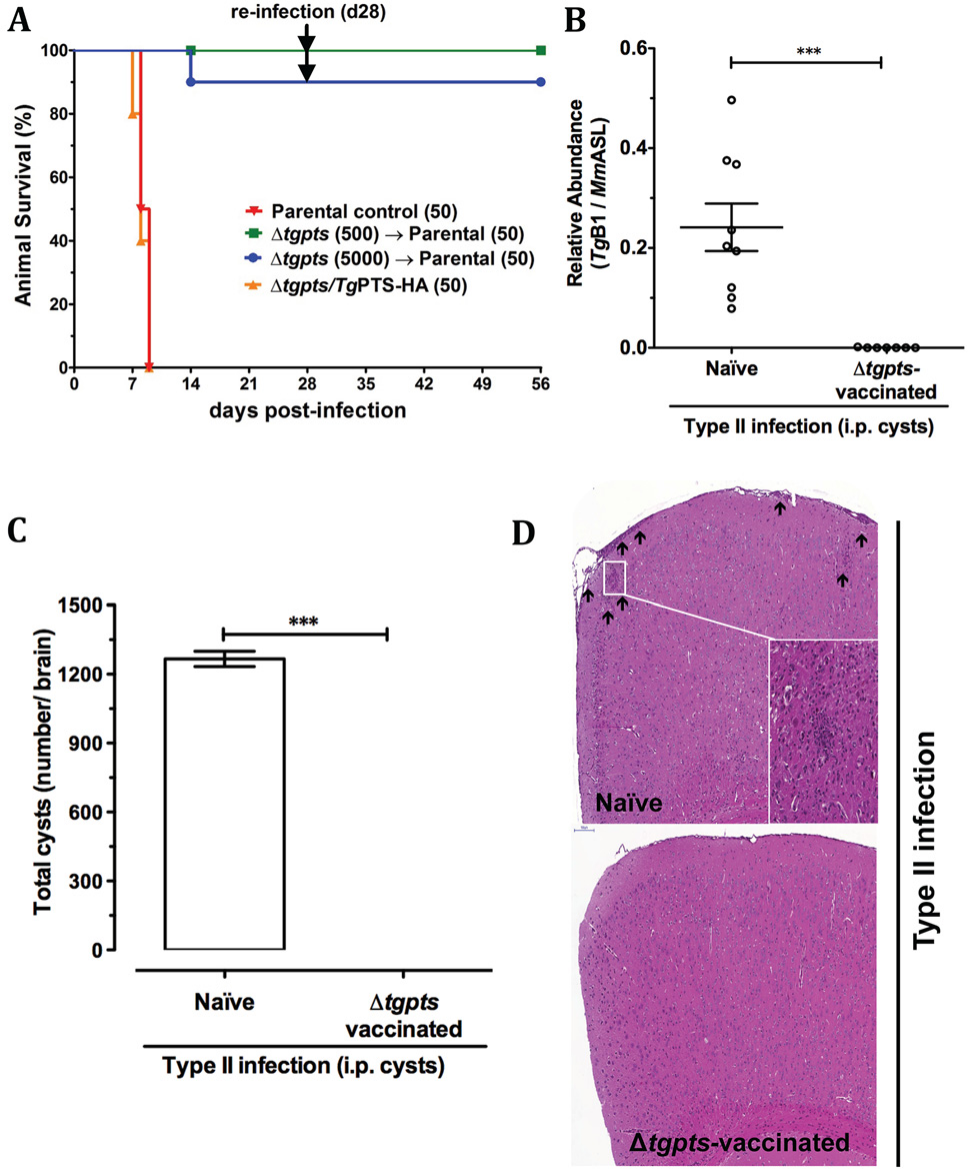
The *Δtgpts* strain is avirulent in mice and provides protection against subsequent acute and chronic toxoplasmosis. **(A)** Infection of mice with the parental, Δ*tgpts*, and Δ*tgpts/Tg*PTS-HA strains to test the virulence and vaccination potential of the mutant against acute infection. Naïve (C57BL/6J) animals were infected either with the RHΔ*ku80-hxgprt* ^*-*^ (50), Δ*tgpts* (500, 5,000), or Δ*tgpts/Tg*PTS-HA (50) strains by intraperitoneal route and monitored for 28 d (*n* = 2–3 experiments, each with 3–5 mice). Animals surviving the Δ*tgpts* infection were reinfected with a lethal dose of the parental strain and examined for additional 4 wk. A control group of naïve mice (*n* = 4) was also infected with the same inoculum. **(B–C)** Infection of the Δ*tgpts*-immunized animals with a cyst-forming strain of *T*. *gondii*. C57BL/6J mice were initially infected with the Δ*tgpts* parasites (500) for 4 wk and subsequently challenged with 3 cysts of the type II (ME49) strain. A control group of naïve mice was also infected. Parasite burden in the brain tissue was evaluated either by real-time PCR (panel B) or by microscopic counting of the parasite cysts (panel C). Panel B shows normalized levels of *Tg*B1 (cyst-specific *T*. *gondii* marker) with respect to the mouse reference (*Mm*ASL). The PCR results show the mean ± SEM of 3 assays, each with 3 mice (***p < 0.001). **(D)** Cerebral histopathological alterations in the control naïve and Δ*tgpts*-vaccinated animals infected with the ME49 strain. Arrows indicate inflammatory foci and meningeal thickening in the brain tissue stained by hematoxylin-eosin (*n* = 2 assays, each with 4 mice).

We also explored the prophylactic potential of the Δ*tgpts* mutant. Examination of virulence in a mouse model demonstrated that nearly all animals infected with the Δ*tgpts* mutant survived as opposed to the parental and PTS-complemented strains, both of which were explicitly lethal (Fig 8A). Importantly, all mice enduring the mutant infection became categorically resistant to a subsequent lethal challenge by a hypervirulent type I strain of *T*. *gondii* causing acute toxoplasmosis (Fig 8A). To further expand the therapeutic utility of our strain as a potential vaccine against chronic infection, we challenged the Δ*tgpts*-infected animals with the cyst-forming type II strain. Remarkably, in contrast to naïve animals, the mutant-vaccinated mice showed no signs of chronic stage cysts in their brain tissue (Fig 8B and 8C). In accord, unlike the naïve control mice, we did not observe any inflammatory lesions in the cortex or meninges of the Δ*tgpts*-immunized animals infected with the type II strain (Fig 8D). In brief, these results demonstrate a requirement of PtdThr for the parasite virulence and illustrate the prophylactic potential of a metabolically attenuated whole-cell “vaccine” against acute and chronic toxoplasmosis.

## Discussion

Our data reveal a natural and fairly abundant expression of PtdThr in a widespread pathogen. We also identified a novel enzyme realizing de novo synthesis of PtdThr in *T*. *gondii*. In addition, the work signifies functional speciation of two closely related lipids, i.e., PtdThr and PtdSer. Last but not least, we show a vital physiological role of PTS and PtdThr for the lytic cycle and virulence of *T*. *gondii*, which can be exploited to develop a vaccine against acute as well as chronic toxoplasmosis.

Besides being the building blocks of biological membranes, phospholipids are involved in many other cellular functions. For example, one of the several roles of PtdSer is to regulate calcium signaling and exocytosis that has been recognized for more than three decades in mammalian cells [21,22]. PtdSer controls Ca^2+^-triggered exocytosis by multiple mechanisms, which involve facilitating the binding of membrane-fusion protein machinery, altering the energy for membrane bending, as well as modulation of PLC-mediated IP_3_-dependent Ca^2+^ channels in the ER [23–25]. Further, anionic phospholipids, such as PtdSer, can also restrict Ca^2+^ slippage into the cytosol by sarcolemmal Ca^2+^-ATPase, which in turn increases the ion capture into the ER [26]. In *T*. *gondii*, calcium signaling is well-known to govern the consecutive events of motility, egression, and invasion by regulating exocytosis of specialized parasite organelles, notably micronemes [27,28]. PtdThr as one of the most abundant anionic lipids regulating Ca^2+^ homeostasis is therefore quite conceivable. Indeed, chemically-synthesized PtdThr derivatives are much more potent inducers of mast cell secretion than PtdSer, and the presence of defined acyl chains exerts a maximal exocytosis [29]—both of these findings are consistent with the natural and dominant existence of selected PtdThr species in *T*. *gondii*. It remains also possible that a lack of PtdThr induces adaptive changes in the parasite ER, which consequently impairs the lytic cycle.

The PTS mutant lacking PtdThr showed a balanced increment in PtdSer, which is reversed by genetic complementation. In line, we observed an apparent increase in the level of another major anionic lipid, PtdIns; however, only when PtdSer content was restored to normal in the double mutant deficient in PtdThr (Δ*tgpts*/*Tg*PSS-2HA-DD without Shield1), but not in the Δ*tgpts* strain regardless of Shield1 in cultures (S12B Fig). Such a specific, reversible, and proportionate amplification of two other anionic lipids appears to maintain the net charge and membrane biogenesis but was entirely unable to mend the lytic cycle. It is therefore plausible that parasite has invented or selected PtdThr for realizing the lytic cycle, while satisfying the customary role of lipids in membrane biogenesis. In this context, it is worth stating that the parasite harbors a putative plant-like pathway to make threonine (www.ToxoDB.org), an amino acid otherwise essential for mammalian host cells. Our assays using stable ^13^C isotope of threonine demonstrated de novo synthesis of PtdThr in replicating *T*. *gondii* (S13 Fig). The isotope-labeled lipid accounted for only about 5% of the total PtdThr in the parasite, which implies a rather inefficient import of threonine by intracellular parasites and a dependence on autonomous synthesis to produce this exclusive lipid. A modest labeling of intracellular parasites with ^13^C-threonine resonates with a rather inefficient incorporation of radioactive precursor by extracellular parasites (not shown). Hence, it appears as though *T*. *gondii* has evolved a serine-threonine homeostasis that is quite distinct from its mammalian host.

Going forward, it will be important to define biochemical features of PtdThr-deprived and PtdSer-enriched mutant membranes. It will also be critical to characterize the biophysical properties of PtdThr species and perform high-resolution imaging of fluorescent analogs to determine its distribution in the parasite organelles. Likewise, knowing the exact sites of lipid synthesis using the antibodies against endogenous PSS and PTS proteins should help define the trafficking of PtdSer and PtdThr and their relative importance for calcium homeostasis in *T*. *gondii*. Most such studies, however, demand pure preparations of PtdThr species, fluorescent lipid derivatives and antibodies, which are not available at this point. Nonetheless, having established the genetic origin and functional relevance of PtdThr in a model pathogen provides a strong basis for future research on mechanism, evolution and therapeutic potential of PTS and PtdThr. Curative importance of a metabolically-attenuated strain has also been exemplified before using an uracil-auxotroph strain [30,31]. This work should therefore enable prospective vaccination studies using the attenuated PTS-disrupted strain, particularly against the yet-incurable and more prevalent chronic infections.

In summary, our research demonstrates the natural and abundant synthesis of an exclusive lipid class, PtdThr, in a widespread protozoan parasite, which is synthesized by a unique enzyme evolved from an otherwise universal protein. We also reveal a lipid-mediated regulation of parasite-specific functions, while illustrating an evolutionary paradigm, i.e., adaptive divergence of the related phospholipids. The physiological need of PTS for the parasite makes it an attractive therapeutic and vaccine target.

## Materials and Methods

### Biological Reagents and Resources

All in vivo assays were performed in compliance with the German animal protection laws directed by Landesverwaltungsamt Sachsen-Anhalt, Germany. The RHΔ*ku80-hxgprt* ^*-*^ strain of *T*. *gondii* and the *pLIC-DHFR-2HA-DD* vector were provided by Vern Carruthers (University of Michigan, United States) [32,33]. The plasmid *pTgTUB8-TgDer1-GFP* was obtained from Boris Striepen (University of Georgia, Athens, US). Primary antibodies for localization studies, mouse anti-*Tg*Mic2, mouse anti-*Tg*Rop1, mouse anti-*Tg*Gra5, mouse anti-*Tg*F1B, rabbit anti-*Tg*VP1 and rabbit anti-*Tg*Fd, were provided by David Sibley (Washington University, St. Louis, US), John Boothroyd (Stanford University School of Medicine, Stanford, US), Marie-France Cesbron-Delauw (CNRS-Université Joseph Fourier, Grenoble, France), Peter Bradley (University of California, Los Angeles, US), Silvia Moreno (University of Georgia, Athens, US), and Frank Seeber (Robert-Koch Institute, Berlin, Germany), respectively. Other primary antibodies used in this work, such as mouse anti-*Tg*Sag1, rabbit anti-*Tg*Gap45, and rabbit anti-*Tg*Hsp90 were offered by Jean-Francois Dubremetz (University of Montpellier, France), Dominique Soldati-Favre (University of Geneva, Switzerland), and Sergio Angel (IIB-INTECH, San Martin, Argentina), respectively. Oligonucleotides were purchased from Life Technologies. C57BL/6J mice were acquired from Janvier Labs (Saint Berthevin, France).

### Parasite and Host Cell Cultures

Tachyzoites of the RHΔ*ku80-hxgprt* ^*-*^ strain were propagated in human foreskin fibroblast (HFF) cells in a humidified incubator (37°C, 5% CO_2_). Cells were cultured in Dulbecco's Modified Eagle Medium (DMEM) supplemented with fetal bovine serum (10%), glutamine (2 mM), MEM nonessential amino acids (100 μM each, glycine, alanine, asparagine, aspartic acid, glutamic acid, proline, serine), sodium pyruvate (1 mM), penicillin (100 U/ml), and streptomycin (100 μg/ml). Parasites were usually cultured at a multiplicity of infection (MOI) of 3 every 2–3 d unless stated otherwise. HFF were harvested by trypsinization and grown to confluence in fresh flasks, dishes, or plates as per experimental needs.

### Molecular Cloning and Genetic Manipulation of *T*. *gondii*

Parasite RNA was isolated using Trizol-based extraction method and subsequently reverse-transcribed into first-strand cDNA (Life Technologies). The complete ORFs of *Tg*PSS and *Tg*PTS were amplified from tachyzoite cDNA using PfuUltra II Fusion polymerase (Agilent Technologies, primers in S1 Table). *Tg*PSS and *Tg*PTS with a C-terminal HA-tag were cloned into the *pTgSAG1-UPKO* or *pTgGRA1-UPKO* plasmid at *EcoR*V/*Pac*I or *Nsi*I/*Pac*I sites, respectively and transformed into *E*. *coli* XL-1b (Stratagene) for cloning and vector amplification. The plasmid constructs were transfected into fresh tachyzoites of the RHΔ*ku80-hxgprt* ^*-*^ or its derivative strains (50 μg DNA, ~10^7^ parasites, 2 kV, 50Ω, 25 μF, 250 μs) using a BTX electroporation instrument. Extracellular parasites were suspended in filter-sterile Cytomix (120 mM KCl, 0.15 mM CaCl_2_, 10 mM K_2_HPO_4_/KH_2_PO_4_, 25 mM HEPES, 2 mM EGTA, 5 mM MgCl_2_, pH 7.6) for transfection and selected for drug resistance.

All *UPKO*-based plasmids allowed a targeted insertion of the PSS or PTS expression cassettes at the uracil phosphoribosyltransferase (UPRT) locus. Tachyzoites with a disrupted *UPRT* gene locus were selected using 5 μM of 5-fluorodeoxyuridine (FUDR) [34]. Stable transgenic parasites expressing PSS or PTS were transiently transfected with the *pTgTUB8-TgDer1-GFP* for colocalization studies. Der1 (degradation in ER 1) protein has long been shown to mediate the ER-associated protein degradation in yeast [35], and more recently in *T*. *gondii* [36]. *Tg*Der1-GFP has also been used before to ascertain the localization of other ER proteins [37]. The PTS deletion construct *pTKO-5’COS-HXGPRT-3’COS* contained COS flanking a hypoxanthine-xanthine-guanine-phosphoribosyltransferase (HXGPRT) expression cassette, which enabled transgenic selection by MPA (25 μg/ml) and xanthine (50 μg/ml) [38]. 5’COS (0.9 kb) and 3’COS (0.8 kb) were amplified using the genomic DNA isolated from host-free tachyzoites and cloned at *Not*I/*EcoR*I and *Hpa*I/*Hpa*I sites of the *pTKO* vector, respectively. Plasmid was linearized with *Apa*I prior to transfection. Clonal drug-resistant parasites were isolated by limiting dilution and screened for 5’- and 3’-recombination events at the *TgPTS* gene locus using applicable primers (S1 Table). The PTS-complemented strain (Δ*tgpts/Tg*PTS-HA) was created by transforming the Δ*tgpts* mutant with the *pTgGRA1-UPKO-TgPTS-HA* plasmid using FUDR selection [34].

To make the Δ*tgpts*/*Tg*PSS-2HA-DD strain, the *TgPSS* gene in the Δ*tgpts* mutant was tagged with a C-terminal 2HA-DD epitope by 3’-insertional tagging (3’-IT). A 1.1-kb COS targeting the 3’-end of the *TgPSS* gene was amplified from tachyzoite gDNA and cloned into the *Pac*I-digested vector (*pLIC-DHFR-2HA-DD*) by ligation-independent cloning (LIC, Clontech). The *pLIC-DHFR-TgPSS-2HA-DD-3’IT* construct was linearized by *Nsi*I in the first half of the COS, transfected into the Δ*tgpts* strain and selected by pyrimethamine (1 μM) [39]. The Δ*tgpts/Tg*PSS-2HA-DD strain expressed *Tg*PSS-2HA-DD under the control of endogenous promoter and *Tg*TUB-3’UTR.

### Lytic Cycle Assays

All experiments were set up using fresh syringe-released extracellular tachyzoites. For plaque assays, 100–200 parasites of each strain were used to infect HFF monolayers in six-well plates. Parasitized cells were incubated for 7 d, fixed with cold methanol, and then stained with crystal violet. Plaques were imaged and scored for their sizes and numbers using the ImageJ software (NIH, US). To test the gliding motility, parasites were incubated on BSA (0.01%)-coated coverslips in Hanks Balanced Salt Solution (HBSS) for 15 min at 37°C. Samples were fixed in 4% paraformaldehyde and 0.05% glutaraldehyde (10 min), and then stained with anti-*Tg*Sag1 and Alexa488 antibodies. Motile fractions and trail lengths were quantified using the ImageJ software.

For invasion and egress assays, HFF monolayers cultured on glass coverslips were infected with fresh parasites for 1 hr (MOI, 10) or for 40–72 hr (MOI, 1), respectively [40]. Samples were subsequently fixed with 4% paraformaldehyde and 0.05% glutaraldehyde in PBS (2 min), neutralized by 0.1 M glycine/PBS (5 min), and then blocked in 3% BSA/PBS (30 min). Noninvasive parasites or egressed vacuoles were stained with anti-*Tg*Sag1 antibody (1:1,500, 1 hr) prior to detergent permeabilization. Cells were washed 3x with PBS, permeabilized with 0.2% triton X 100/PBS (20 min), and stained with anti-*Tg*Gap45 antibody (1:3,000, 1 hr) to visualize intracellular parasites. Samples were washed and immunostained with Alexa488 and Alexa594-conjugated antibodies (1:3,000, 1 hr). The number of invaded parasites was deduced by immunostaining with anti-*Tg*Gap45/Alexa594 (red), but not with anti-*Tg*Sag1/Alexa488 (green). The egressed vacuoles were scored directly from the number of vacuoles with *Tg*Sag1-stained parasites.

### Immunofluorescence Localization

Localization of epitope-tagged proteins was performed by immunofluorescence assays. The method was essentially the same as described for invasion assays except for that samples were permeabilized prior to incubation with antibodies. A panel of organelle-specific antibodies (*Tg*Mic2 for micronemes, 1:1,000; *Tg*Rop1 for rhoptries, 1:1,000; *Tg*Gra5 for dense granules, 1:500; *Tg*F1B for mitochondrion, 1:1,000; *Tg*Fd for apicoplast, 1:500; *Tg*VP1 for acidocalcisomes/plant-like vacuole, 1:500) was used together with anti-HA antibody (1:5,000; Sigma-Aldrich, Germany) to assess localizations of epitope-tagged PSS and PTS proteins. Images were acquired using ApoTome microscope (Zeiss, Germany).

### Functional Expression in *E*. *coli*

The M15/pREP4 strain was transformed with the empty *pQE60* expression vector (Qiagen), *pQE60*-*Tg*PTS, *pQE60*-*Tg*PSS, or *pQE60*-*At*PSS [17] constructs and cultured in Luria-Broth medium supplied with ampicillin (100 mg/L) and kanamycin (50 mg/L). Protein expression was induced by 1 mM IPTG at 25°C in overnight cultures containing 5 mM threonine or serine, followed by a 4 hr incubation at 37°C. Lipids were isolated and separated by one-dimensional TLC in chloroform/methanol/acetate (130:50:20) and visualized by ninhydrin staining.

### Lipid Extraction, TLC, and Phosphorus Quantification

Parasites were syringe-released from infected HFF (MOI, 3; 42–48 hrs of infection) and passed twice through 23G and 27G needles. Host debris was removed by filtering the parasite suspension through a 5 μm filter (Merck Millipore, Germany). Cell pellets (0.5-1x10^8^ parasites) were resuspended in 0.4 ml of PBS and lipids were extracted according to Bligh-Dyer [41]. Briefly, 0.5 ml chloroform and 1 ml methanol were mixed to the samples, which were allowed to stand for 30 min and centrifuged (2,000 g, 5 min). The supernatant was transferred to a glass tube followed by addition of chloroform and 0.9% KCl (1 ml each). Samples were mixed, centrifuged and the lower chloroform phase containing lipids was transferred to a conical glass tube. Samples were stored at −20°C in the airtight glass tubes flushed with nitrogen gas. Lipids were resolved by two-dimensional TLC on silica gel 60 plates (Merck) using chloroform/methanol/ammonium hydroxide (65:35:5) and chloroform/acetic acid/methanol/water (75:25:5:2.2) as the solvents for the first and second dimensions, respectively. They were visualized by staining with iodine vapors and identified based on their migration with authentic standards (Avanti Lipids). The major iodine-stained phospholipid bands were scraped off the silica plate, and quantified by chemical phosphorus assay, as described elsewhere [42].

### Lipidomics Analyses

Total lipids (0.5–1 x 10^8^ tachyzoites) were fractionated on chloroform-equilibrated silica 60 columns. Neutral lipids were eluted by acetone washing of the column. Phospholipids were subsequently eluted in 5 column-volumes of chloroform/methanol/water (1:9:1). Each lipid fraction was collected, dried under nitrogen stream at 37°C, and stored at −20°C for downstream assays. Internal standard PtdCho (44:2) was mixed with extracted lipids to calibrate the recovery yield of major lipids. 10−20 μl aliquots of phospholipid extract in chloroform/methanol (1:1) were introduced onto a HILIC column (Kinetex, 2.6 μm) at a flow rate of 1 ml/min to resolve different phospholipid classes, essentially as described elsewhere [43]. Column effluent was introduced into either a 4,000 Q-TRAP mass spectrometer (AB Sciex, Framingham, MA) or LTQ-XL (Thermo Scientific, Waltham, MA), and analyzed in the negative ion mode using electrospray ionization. Data were processed using the proprietary software of the respective instrument manufacturers. Lipidomics data reported in this work have been deposited in the Dryad repository [44]: http://dx.doi.org/10.5061/dryad.564sc

### In Vivo Parasite Infection and Cerebral Histopathology

C57BL/6J mice were infected with extracellular tachyzoites of the RHΔ*ku80-hxgprt* ^*-*^ (parental), Δ*tgpts* or Δ*tgpts/Tg*PTS-HA strains. Parasites for in vivo infections were propagated in HFF cells. Fresh host-free tachyzoites were syringe-released after 40 hr of infection, filtered (5 μm), and then injected via intraperitoneal (i.p.) route (50 parasites of the parental and Δ*tgpts/Tg*PTS-HA strains; 5 x 10^2^ or 5 x 10^3^ of Δ*tgpts* strain). Animals were monitored for mortality and morbidity 3 times a day over a period of 4 wk. An inoculum of 50 parental tachyzoites (type I) was used to challenge the Δ*tgpts*-immunized animals, which were monitored for additional 4 wk.

Cysts were harvested from the brains of female NMRI mice infected with *T*. *gondii* of the ME49 strain 5 to 6 months earlier (i.p.), as described before [45]. The Δ*tgpts*-vaccinated mice (500 parasites) were challenged with the type II parasites (ME49, 3 cysts i.p. in 200 μl) 4 wk after the primary infection. A control group of naïve animals was also included. Parasite burden in the mouse brain was estimated by counting cysts and semiquantitative real-time PCR following another 4 wk of infection with the ME49 strain. Brain tissue was mechanically homogenized in 1 ml sterile PBS and cysts were counted using a light microscope. For qPCR, perfused brain tissue samples were snap-frozen and stored at −80°C [46]. 30 mg tissue was used to purify nucleic acids (QIAgen kit). FastStart Essential DNA Green Master (Roche, Germany) was mixed with genomic DNA (90 ng) in triplicate reactions, which were developed in a LightCycler 480 Instrument II (Roche, Germany). The parasite burden (target: *Tg*B1 gene) was estimated relative to mouse (reference: argininosuccinate lyase, *Mm*ASL). Primers for the *Tg*B1 and *Mm*ASL genes are listed in S1 Table. For cerebral histopathology, brain tissues isolated from infected animals were immersed in 4% paraformaldehyde for several days. Samples were embedded in paraffin, sliced into 4-μm thick sections, deparaffinized and then stained with hematoxylin-eosin stain, as described elsewhere [47]. Slides were developed using the Bond polymer refine detection kit (Menarini/Leica, Germany). Tissue sections were scanned at 230 nm resolution using a MiraxMidi Scanner (Zeiss MicroImaging GmbH, Germany) [48].

## Supporting Information

S1 Data Excel spreadsheet containing underlying numerical data and statistical analyses for Figs 1A, 5B–5E, 6B and 6C, 7B and 7C, 8A–8C, S1A, S7, S8B, S9A and S9B and S12A and S12B Figs. (XLSX)

S1 Fig PtdThr is a major phospholipid in *T*. *gondii*. **(A)** HPLC profile of threonine obtained by hydrolysis of X1-lipid from extracellular tachyzoites (10^7^). Detection and quantification was achieved by multiple-reaction-monitoring (MRM) MS of threonine decarboxylation (transition, 120/74 Da). **(B)** Two-dimensional TLC of lipids from tachyzoites (10^8^) showing major iodine-stained phospholipids. Lipids were identified by their migration patterns in comparison to authentic phospholipid standards except for PtdThr, for which no commercial standard is available. **(C)** Chemical identity of PtdThr by MS analysis. TLC-resolved X1 band from panel B was confirmed as PtdThr by fragmentation pattern and *m/z* ratios.
(TIFF)

S2 Fig Human foreskin fibroblast cells do not contain detectable amounts of phosphatidylthreonine. **(A)** Liquid chromatography-mass spectrometry (LC-MS) elution profile showing the retention times and peak intensities of phospholipids isolated from human fibroblasts. **(B)** MS analysis of the indicated fraction revealing the prevalent occurrence of PtdSer species and a complete lack of detectable PtdThr species. Fibroblast lipids were detected in the negative ionization mode, as described for the parasite lipids.
(TIFF)

S3 Fig Orthologs of PtdThr synthase are present in selected free-living and parasitic protists but absent in most other organisms. Phylogenetic analysis of the orthologs of PTS and PSS from distinct organisms shows an early divergence of the two enzymes. *Tg*PSS (ToxoDB: TGGT1_261480) clusters with the mainstream PSS clade that also comprises other parasite orthologs. In contrast, *Tg*PTS (ToxoDB: TGGT1_273540) segregates with selected parasitic (*Eimeria*, *Neospora*, *Phytophtora*) and free-living (*Perkinsus*) chromalveolates. Colored circles signify bootstrap values. Sequences for performing phylogenetic analysis (www.phylogeny.fr) were obtained from the NCBI (www.ncbi.nlm.nih.gov) and parasite databases (www.ToxoDB.org). Accession numbers are indicated next to the sequence. NCBI accession IDs for *Tg*PTS and *Tg*PSS are KJ026547 and KJ026548, respectively.
(TIFF)

S4 Fig PtdThr synthase from *T*. *gondii* harbors multiple substitutions in the catalytic domain of an otherwise universal base-exchange-type PtdSer synthase. **(A)** Secondary structure and membrane topology of *Tg*PTS, as predicted by SOSUI program (http://bp.nuap.nagoya-u.ac.jp/sosui). **(B)** Amino acid sequence alignment of PSS and PTS from *T*. *gondii* with orthologs from indicated organisms. The diamond and arrow signs specify the residues contributing to the PSS activity and to substrate binding, respectively. Other conserved residues in PSS proteins show distinct substitutions in PTS orthologs (colored boxes). Gray bar under the alignment denotes the transmembrane domain.
(TIFF)

S5 Fig Immunofluorescence costaining of *Tg*PTS-HA with organelle-specific markers. Transgenic parasites ectopically expressing *Tg*PTS-HA under the control of the *TgGRA1* promoter and 3’UTR at the UPRT locus were generated by FUDR selection. Primary antibodies recognizing the Mic2, Rop1, Gra5, F1B, Fd, and VP1 proteins were used to visualize micronemes, rhoptries, dense granules, mitochondrion, apicoplast, and acidocalcisomes/plant-like vacuole, respectively. Rop1 and Fd staining often showed diffused and high background. No crossfluorescence was observed across the two color channels. Note that majority of the red (anti-hemagglutinin [HA]) staining in the merged panel did not colocalize with any of the organelle markers examined here.
(TIFF)

S6 Fig Targeted disruption of *Tg*PTS does not alter the expression of adjacent genes. **(A)** A genome browser view of *Tg*PTS (TGGT1_273540) on the chromosome VIII of *T*. *gondii* (www.ToxoDB.org). **(B)** ORF-specific PCR of TGGT1_273550 and TGGT1_273530 amplified from total RNA (100 ng) of the parental, Δ*tgpts* mutant and PTS-complemented strains.
(TIFF)

S7 Fig The *Δtgpts* mutant lacks PtdThr and shows a corresponding increase in PtdSer content. Total parasite lipids (0.8–1 x 10^8^ tachyzoites) were resolved by two-dimensional TLC in chloroform/methanol/ammonium hydroxide (65:35:5) and chloroform/acetic acid/methanol/water (75:25:5:2.2) and visualized by iodine vapor staining. Individual lipid bands were scraped off the TLC plate and subjected to chemical phosphorus assay (*n* = 4 assays, *p < 0.05).
(TIFF)

S8 Fig Loss of PtdThr in the *Δtgpts* mutant upregulates PtdSer synthesis in a reversible manner. **(A)** Autoradiography of TLC-resolved parasite lipids following metabolic labeling with radioactive serine. Fresh extracellular parasites of the indicated strains were labeled with ^14^C-serine (2 μCi, 100 μM, 2 hr, 37°C, 5 x 10^7^ parasites). Solvent system used for TLC was chloroform/ethanol/water/triethylamine (30:35:7:35). Lipid bands were identified by migration with authentic standards. **(B)** Radiolabeled lipid bands from panel A were scraped for scintillation counting to determine the usage of serine into PtdSer and PtdEtn (mean ± SEM; *n* = 5 assays; **, *p* < 0.01; ***, *p* < 0.001).
(TIFF)

S9 Fig Base-exchange activity of PTS is required for an optimal growth of *T*. *gondii*. **(A–B)** Growth of the indicated parasite strains, as deduced by plaque assays. The decreased size **(A)** and number **(B)** of plaques formed by the Δ*tgpts* mutant were significantly recovered by expression of a functional (wild-type) *Tg*PTS-HA, but not by a catalytically-dead (*Tg*PTS_(ΔECWWD)_-myc) isoform. In total, 50–130 plaques of each strain from 4 assays were analyzed (mean ± SEM; *p < 0.05, **p < 0.01, ***p < 0.001).
(TIFF)

S10 Fig Conditional regulation of *Tg*PSS protein levels by a C-terminal destabilization domain (DD) in the *Δtgpts* mutant. **(A)** Scheme showing the 3’-tagging of the *TgPSS* gene with the 2HA-DD epitope in the Δ*tgpts* strain. Prior to transfecting parasites, the construct was linearized at the *Nsi*I site to enable single homologous recombination at the 3’-end of the gene without perturbing the promoter sequence. Stable transgenic parasites (Δ*tgpts*/*Tg*PSS-2HA-DD) were generated by pyrimethamine selection. **(B)** Immunofluorescence images illustrating staining of *Tg*PSS-2HA-DD with *Tg*Der1-GFP (ER marker), and its regulation in the Δ*tgpts*/*Tg*PSS-2HA-DD strain. Parasites were cultured in Shield1 (0.5 μM, 24 hrs) prior to immunostaining. **(C)** Conditional regulation of *Tg*PSS-2HA-DD by Shield1, as confirmed by immunoblot analyses. Parasitized cells were cultured in 0.5 μM Shield1 for 48 hr prior to detection with anti-HA antibody (*Tg*Hsp90, loading control). As expected, the anti-HA signal is absent in the untreated control samples in panels B and C. The absence of red staining in panel B (without Shield1) also precludes any “bleeding effect” from green to red channel.
(TIFF)

S11 Fig Immunofluorescence imaging of *Tg*PSS-2HA-DD with organelle markers. Stable transgenic parasites expressing *Tg*PSS-2HA-DD under the control of its own promoter and *TgTUB8*-3’UTR were generated by 3’-insertional tagging of the gene, as described in S10 Fig. Cultures were treated with 0.5 μM Shield1 for 24 hr prior to immunostaining to visualize the fusion protein. Staining of Mic2, Rop1, Gra5, F1B, Fd, and VP1 proteins represents micronemes, rhoptries, dense granules, mitochondrion, apicoplast and acidocalcisomes/plant-like vacuole, respectively. Samples stained with anti-Rop1 and anti-Fd antibodies exhibited diffused and high background fluorescence, occasionally transecting with anti-HA. Most of the HA signal in the merged image however did not colocalize with any organelles except for mitochondrion and acidocalcisomes/plant-like vacuole, often superimposing ER extensions.
(TIFF)

S12 Fig Conditional destabilization of *Tg*PSS activity restores a normal PtdSer synthesis and lipid content in the *Δtgpts* strain. (**A)** Incorporation of ^14^C-serine into total lipid fraction of host-free parasites precultured during the intracellular phase without or with Shield1 (0.5 μM, 24 hrs). Labeling of parasites was done, as described in Fig 7A (mean ± SEM, *n* = 4 assays; *p < 0.05, **p < 0.01). **(B)** Quantification of lipid-phosphorus in the indicated parasites strains. Lipids (0.8–1 x 10^8^ tachyzoites) were resolved by two-dimensional TLC and subjected to lipid-phosphorus assay (mean ± SEM of 3 assays; *p < 0.05). The data in panels A–B also confirm the catalytic function of *Tg*PSS in *T*. *gondii*.
(TIFF)

S13 Fig Intracellular parasites can synthesize PtdThr using free threonine in cultures. The parental parasites (RHΔ*ku80-hxgprt* ^*-*^) were grown in HFF monolayers supplied with 0.4 mM ^13^C-threonine for 2 d. Lipids from syringe-released purified parasites were subjected to MS/MS analyses. Unlabeled samples were also analyzed to illustrate the natural abundance of ^13^C. The transitions 854.5–753.5 and 854.5–749.5 represent the neutral losses of ^12^C_4_-Thr (nl101) and ^13^C_4_-Thr (nl105), respectively, in the PtdThr peak (*m/z* 854.5, 40:5, 4x^13^C). ^13^C_1-4_-Thr indicates that all carbons are labeled in the threonine moiety (peak 749.5) of samples incubated with the stable isotope but not in the control, where the natural abundance of 4x labeled threonine is basically zero (no peak at 749.5 in unlabeled sample).
(TIFF)

S1 Table Oligonucleotides used in this study. (PDF)

## Abbreviations

COS: crossover sequence
DD: degradation domain
ER: endoplasmic reticulum
FUDR: 5-fluorodeoxyuridine
HA: hemagglutinin
HPLC: high performance liquid chromatography
HXGPRT: hypoxanthine-xanthine-guanine phosphoribosyltransferase
IPTG: isopropyl β-D-1-thiogalactopyranoside
LC-MS: liquid chromatography/mass spectrometry
MPA: mycophenolic acid
MRM: multiple-reaction-monitoring
MS: mass spectrometry
ORF: open reading frame
PCR: polymerase chain reaction
PEtn-Cer: phosphoethanolamine-ceramide
PSS: phosphatidylserine synthase
PtdCho: phosphatidylcholine
PtdEtn: phosphatidylethanolamine
PtdGro: phosphatidylglycerol
PtdIns: phosphatidylinositol
PtdSer: phosphatidylserine
PtdThr: phosphatidylthreonine
PTS: phosphatidylthreonine synthase
SEM: standard error of the mean
SM: sphingomyelin
TLC: thin layer chromatography
UPRT: uracil phosphoribosyltransferase
VP1: vacuolar proton pyrophosphatase 1
XA: xanthine
