# Supplementary figures and images for: Phosphatidylthreonine and Lipid-Mediated Control of Parasite Virulence
Source: PLoS Biol. 2015 Nov 13;13(11):e1002288. doi: 10.1371/journal.pbio.1002288 (PMC4643901; doi:10.1371/journal.pbio.1002288)

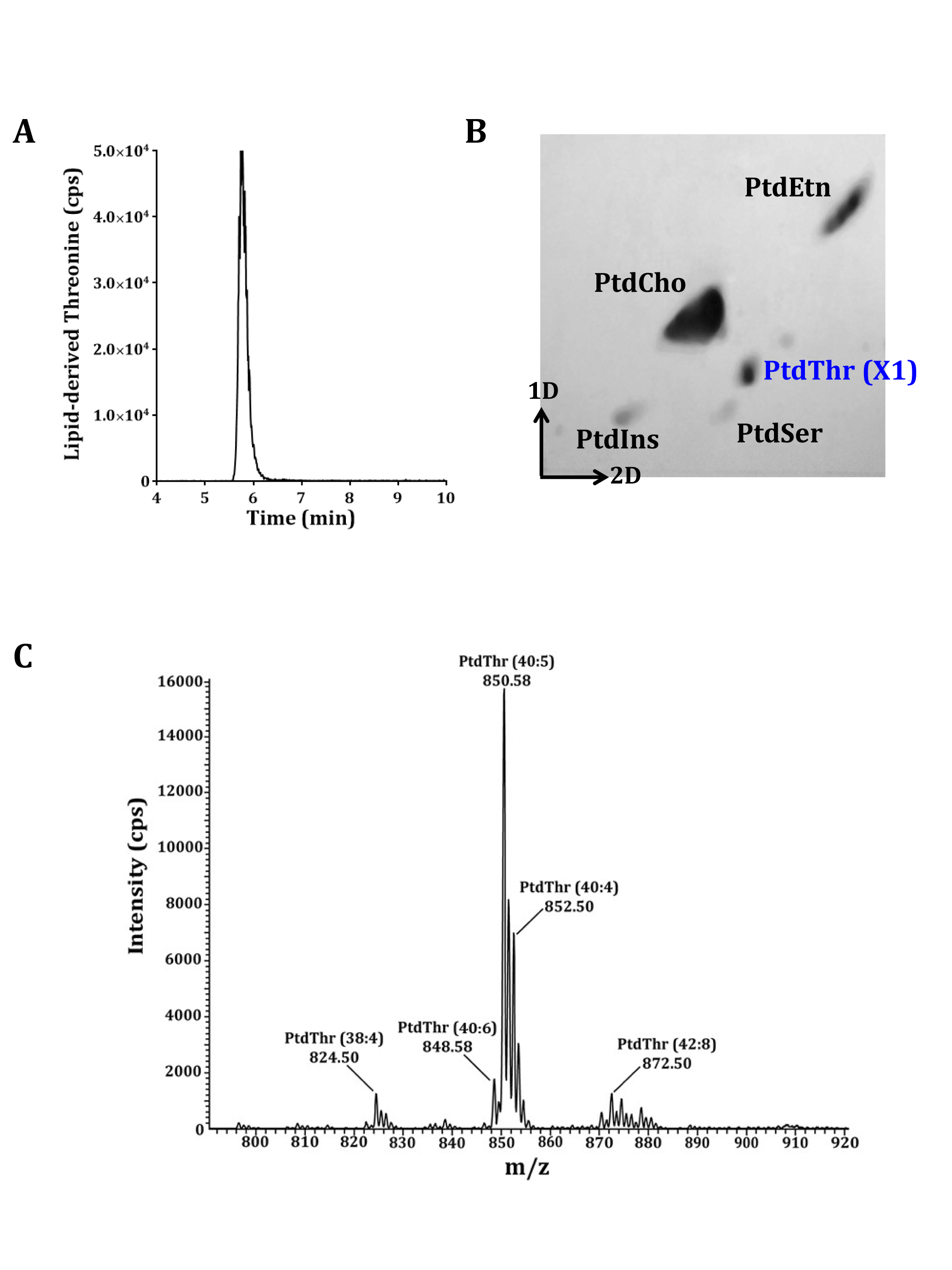

Supplement: S1 Fig — (A) HPLC profile of threonine obtained by hydrolysis of X1-lipid from extracellular tachyzoites (107). Detection and quantification was achieved by multiple-reaction-monitoring (MRM) MS of threonine decarboxylation (transition, 120/74 Da). (B) Two-dimensional TLC of lipids from tachyzoites (108) showing major iodine-stained phospholipids. Lipids were identified by their migration patterns in comparison to authentic phospholipid standards except for PtdThr, for which no commercial standard is available. (C) Chemical identity of PtdThr by MS analysis. TLC-resolved X1 band from panel B was confirmed as PtdThr by fragmentation pattern and m/z ratios. (TIFF) [file pbio.1002288.s002.TIFF]

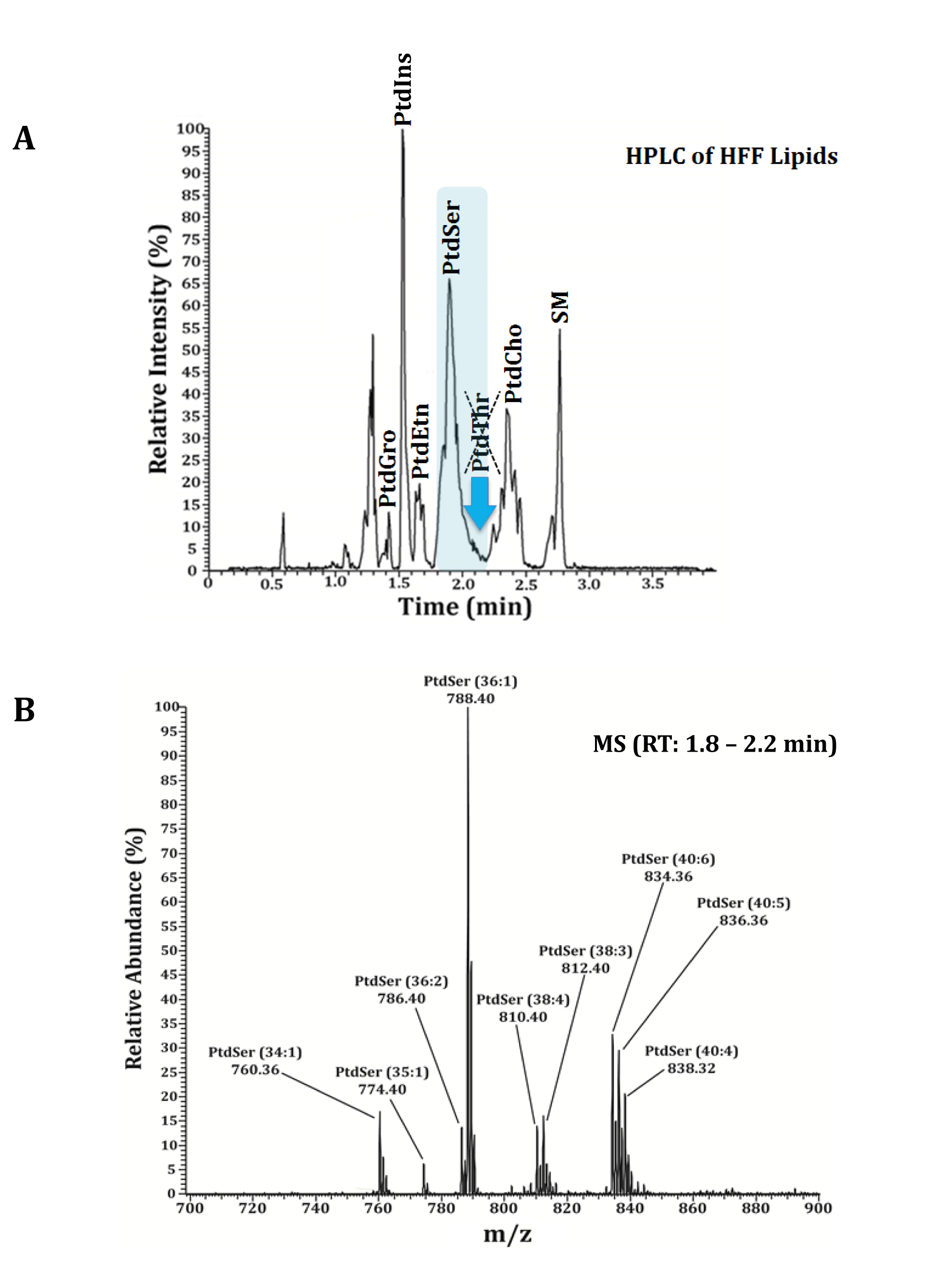

Supplement: S2 Fig — (A) Liquid chromatography-mass spectrometry (LC-MS) elution profile showing the retention times and peak intensities of phospholipids isolated from human fibroblasts. (B) MS analysis of the indicated fraction revealing the prevalent occurrence of PtdSer species and a complete lack of detectable PtdThr species. Fibroblast lipids were detected in the negative ionization mode, as described for the parasite lipids. (TIFF) [file pbio.1002288.s003.TIFF]

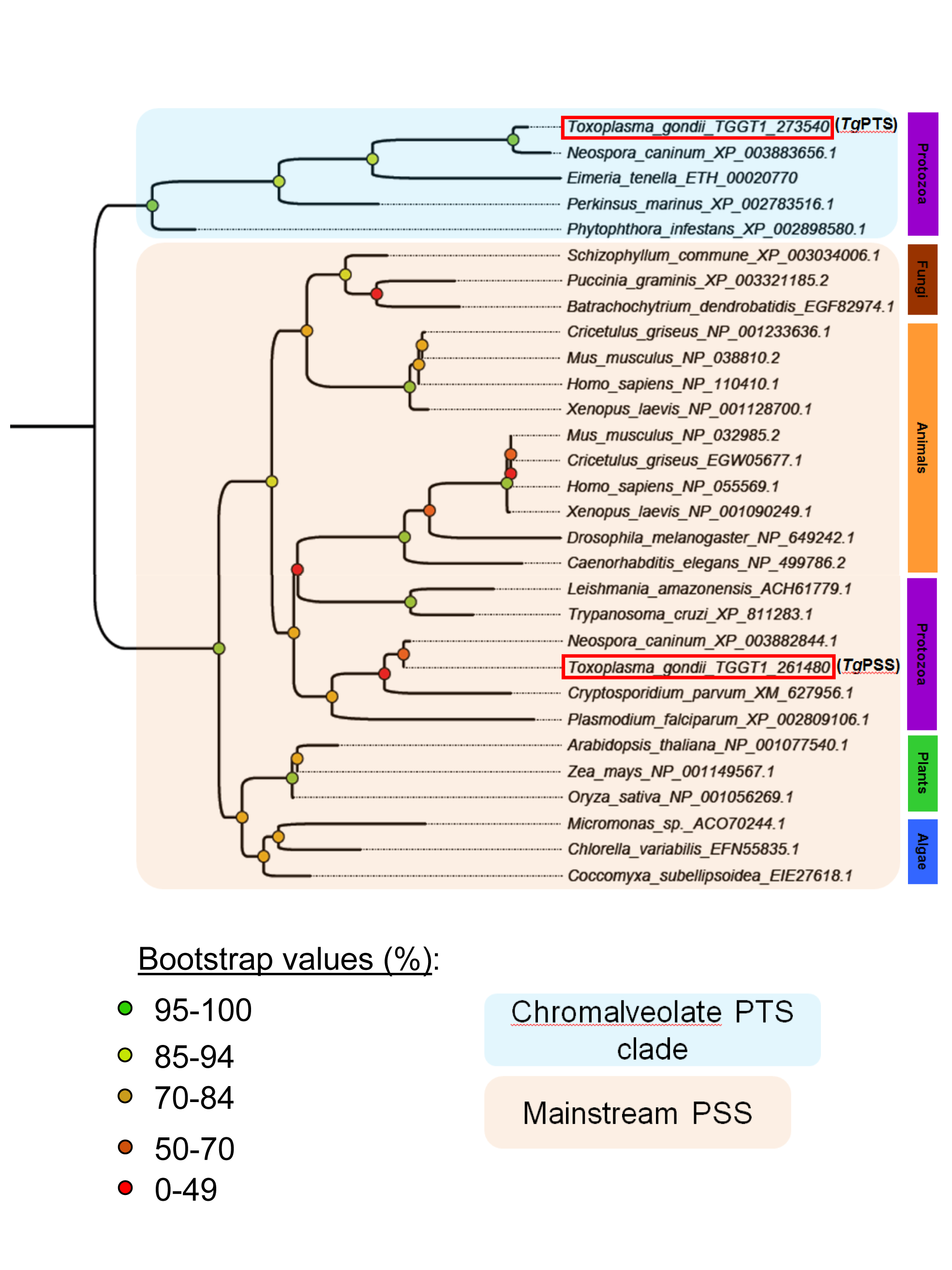

Supplement: S3 Fig — Phylogenetic analysis of the orthologs of PTS and PSS from distinct organisms shows an early divergence of the two enzymes. TgPSS (ToxoDB: TGGT1_261480) clusters with the mainstream PSS clade that also comprises other parasite orthologs. In contrast, TgPTS (ToxoDB: TGGT1_273540) segregates with selected parasitic (Eimeria, Neospora, Phytophtora) and free-living (Perkinsus) chromalveolates. Colored circles signify bootstrap values. Sequences for performing phylogenetic analysis (www.phylogeny.fr) were obtained from the NCBI (www.ncbi.nlm.nih.gov) and parasite databases (www.ToxoDB.org). Accession numbers are indicated next to the sequence. NCBI accession IDs for TgPTS and TgPSS are KJ026547 and KJ026548, respectively. (TIFF) [file pbio.1002288.s004.TIFF]

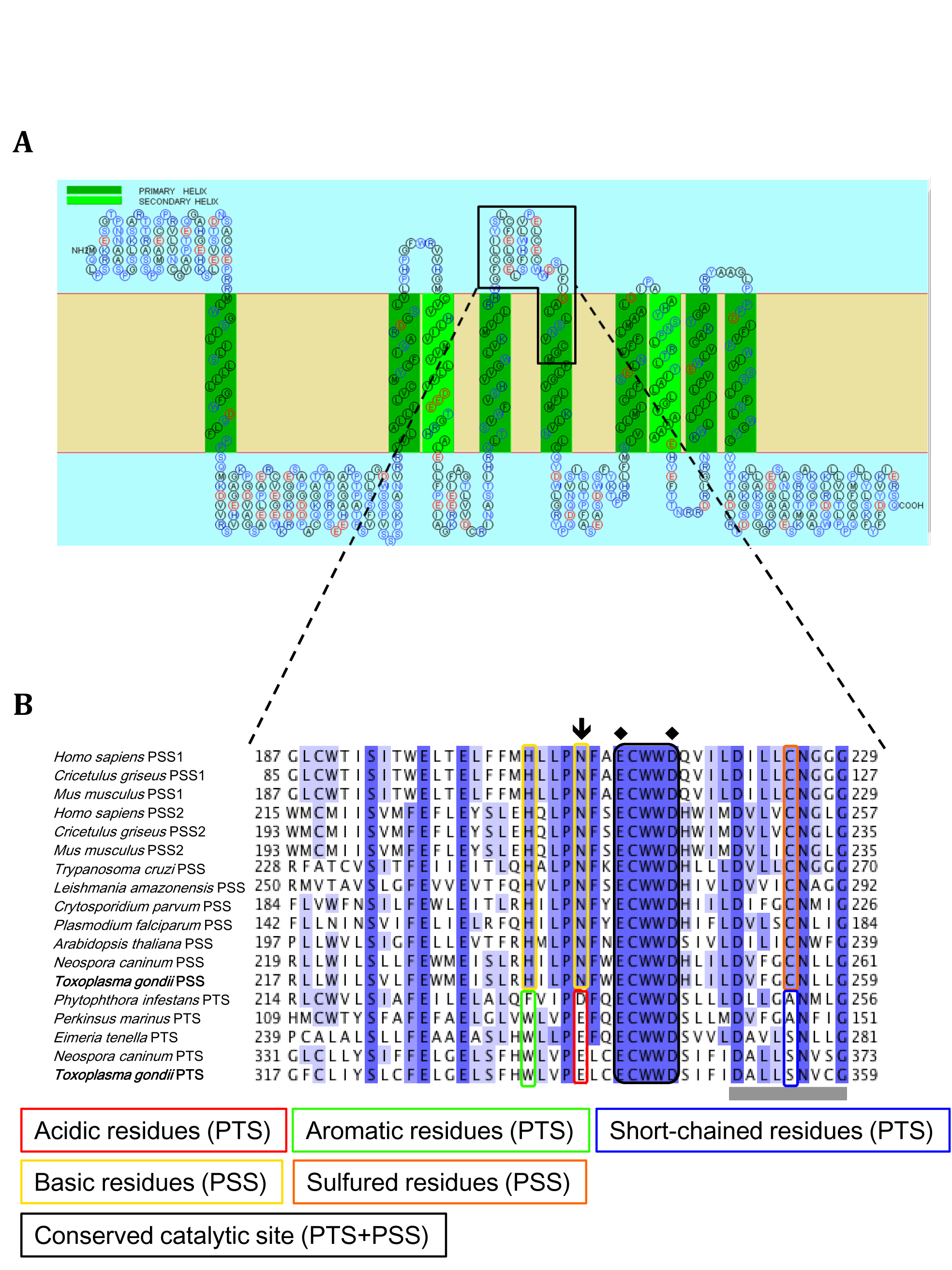

Supplement: S4 Fig — (A) Secondary structure and membrane topology of TgPTS, as predicted by SOSUI program (http://bp.nuap.nagoya-u.ac.jp/sosui). (B) Amino acid sequence alignment of PSS and PTS from T. gondii with orthologs from indicated organisms. The diamond and arrow signs specify the residues contributing to the PSS activity and to substrate binding, respectively. Other conserved residues in PSS proteins show distinct substitutions in PTS orthologs (colored boxes). Gray bar under the alignment denotes the transmembrane domain. (TIFF) [file pbio.1002288.s005.TIFF]

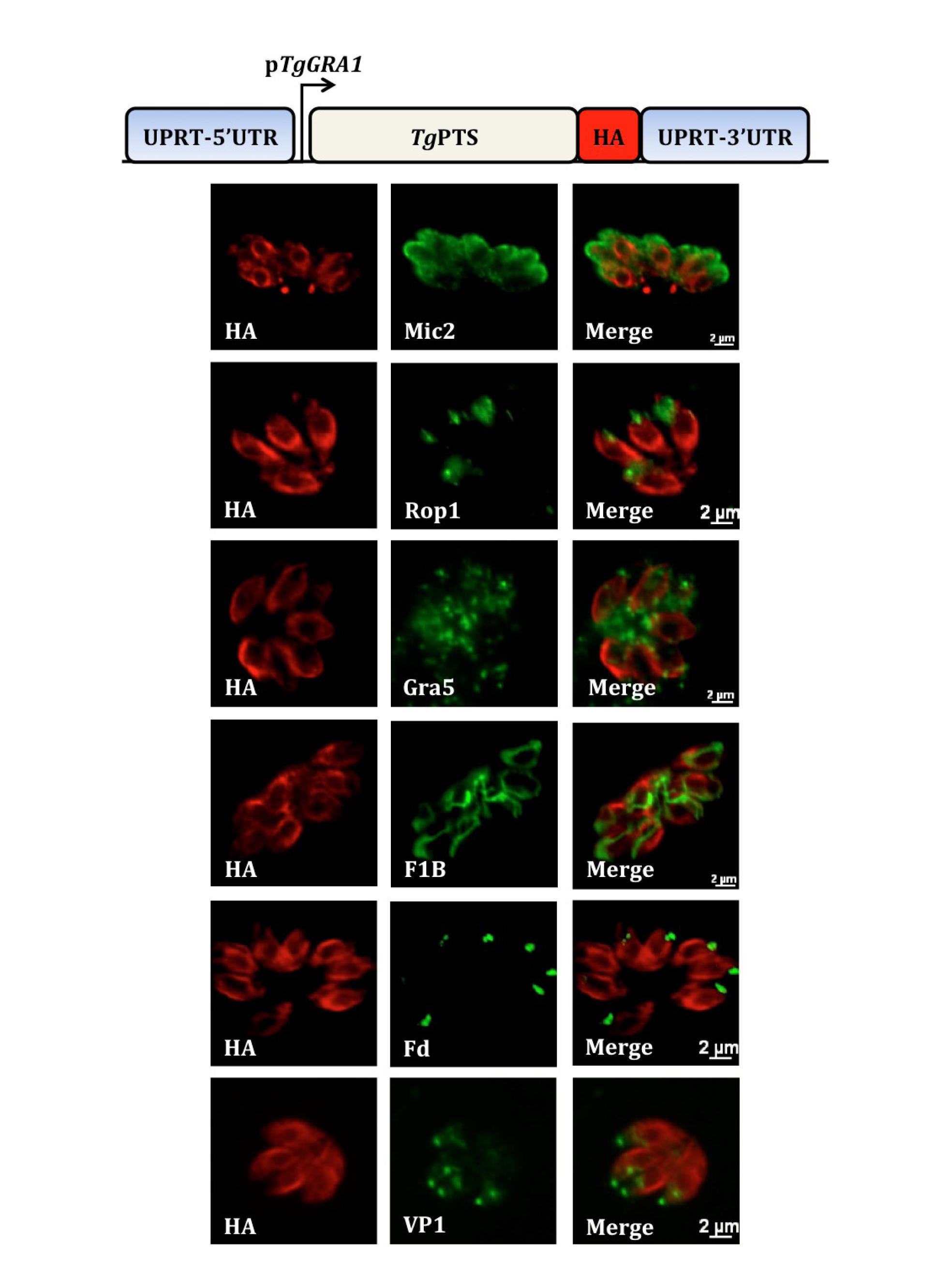

Supplement: S5 Fig — Transgenic parasites ectopically expressing TgPTS-HA under the control of the TgGRA1 promoter and 3’UTR at the UPRT locus were generated by FUDR selection. Primary antibodies recognizing the Mic2, Rop1, Gra5, F1B, Fd, and VP1 proteins were used to visualize micronemes, rhoptries, dense granules, mitochondrion, apicoplast, and acidocalcisomes/plant-like vacuole, respectively. Rop1 and Fd staining often showed diffused and high background. No crossfluorescence was observed across the two color channels. Note that majority of the red (anti-hemagglutinin [HA]) staining in the merged panel did not colocalize with any of the organelle markers examined here. (TIFF) [file pbio.1002288.s006.TIFF]

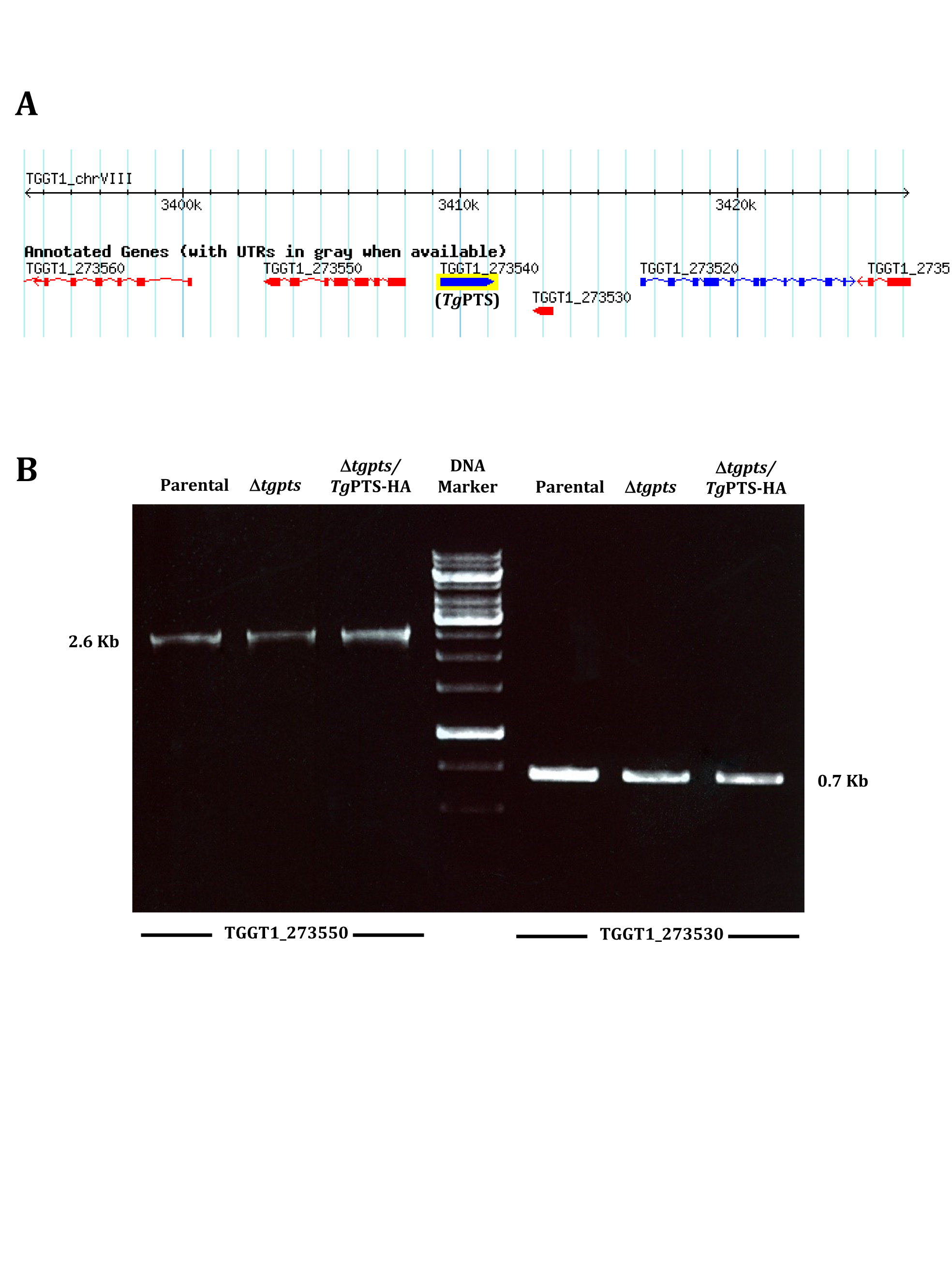

Supplement: S6 Fig — (A) A genome browser view of TgPTS (TGGT1_273540) on the chromosome VIII of T. gondii (www.ToxoDB.org). (B) ORF-specific PCR of TGGT1_273550 and TGGT1_273530 amplified from total RNA (100 ng) of the parental, Δtgpts mutant and PTS-complemented strains. (TIFF) [file pbio.1002288.s007.TIFF]

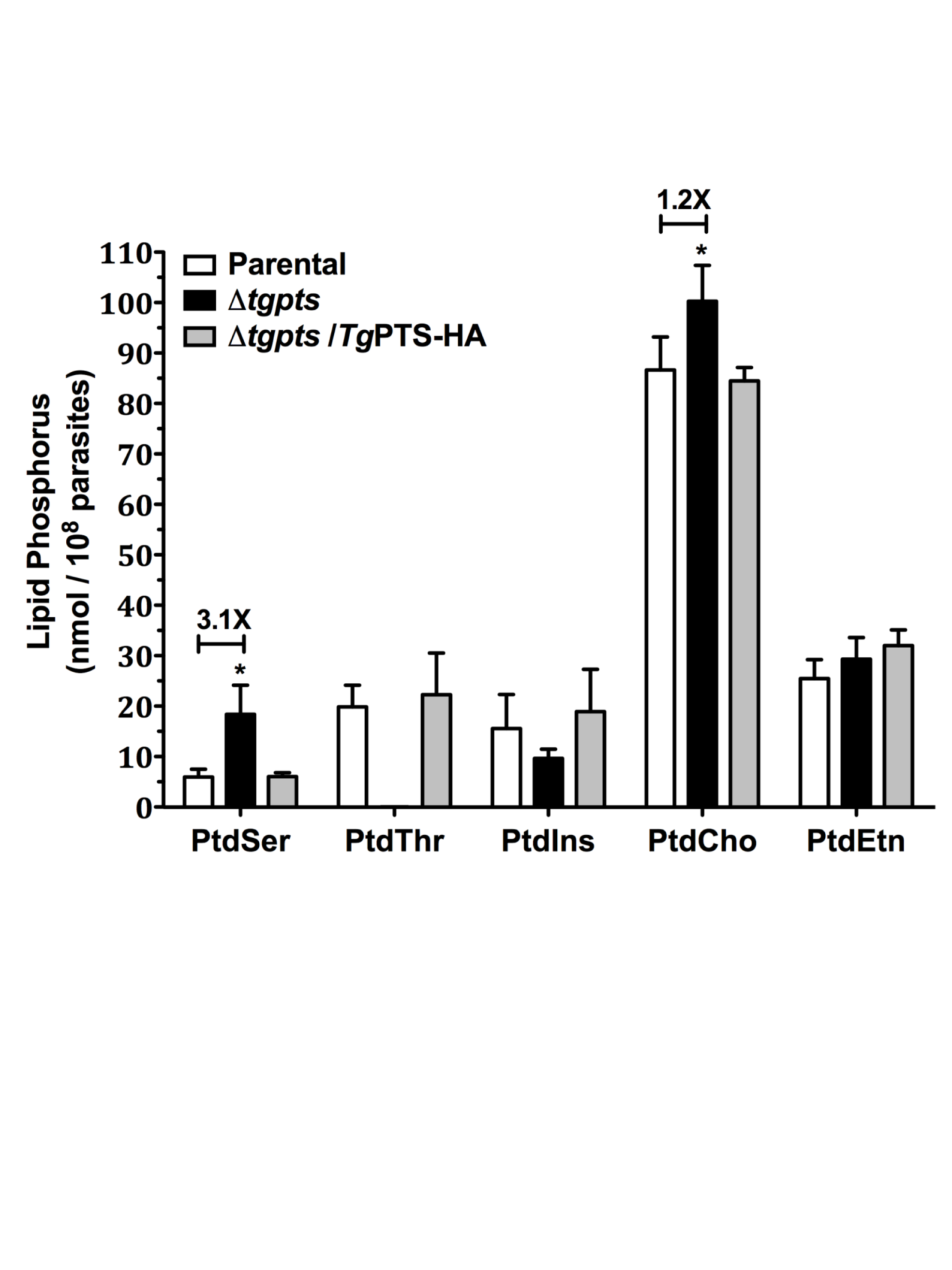

Supplement: S7 Fig — Total parasite lipids (0.8–1 x 10^8^ tachyzoites) were resolved by two-dimensional TLC in chloroform/methanol/ammonium hydroxide (65:35:5) and chloroform/acetic acid/methanol/water (75:25:5:2.2) and visualized by iodine vapor staining. Individual lipid bands were scraped off the TLC plate and subjected to chemical phosphorus assay (n = 4 assays, *p < 0.05). (TIFF) [file pbio.1002288.s008.TIFF]

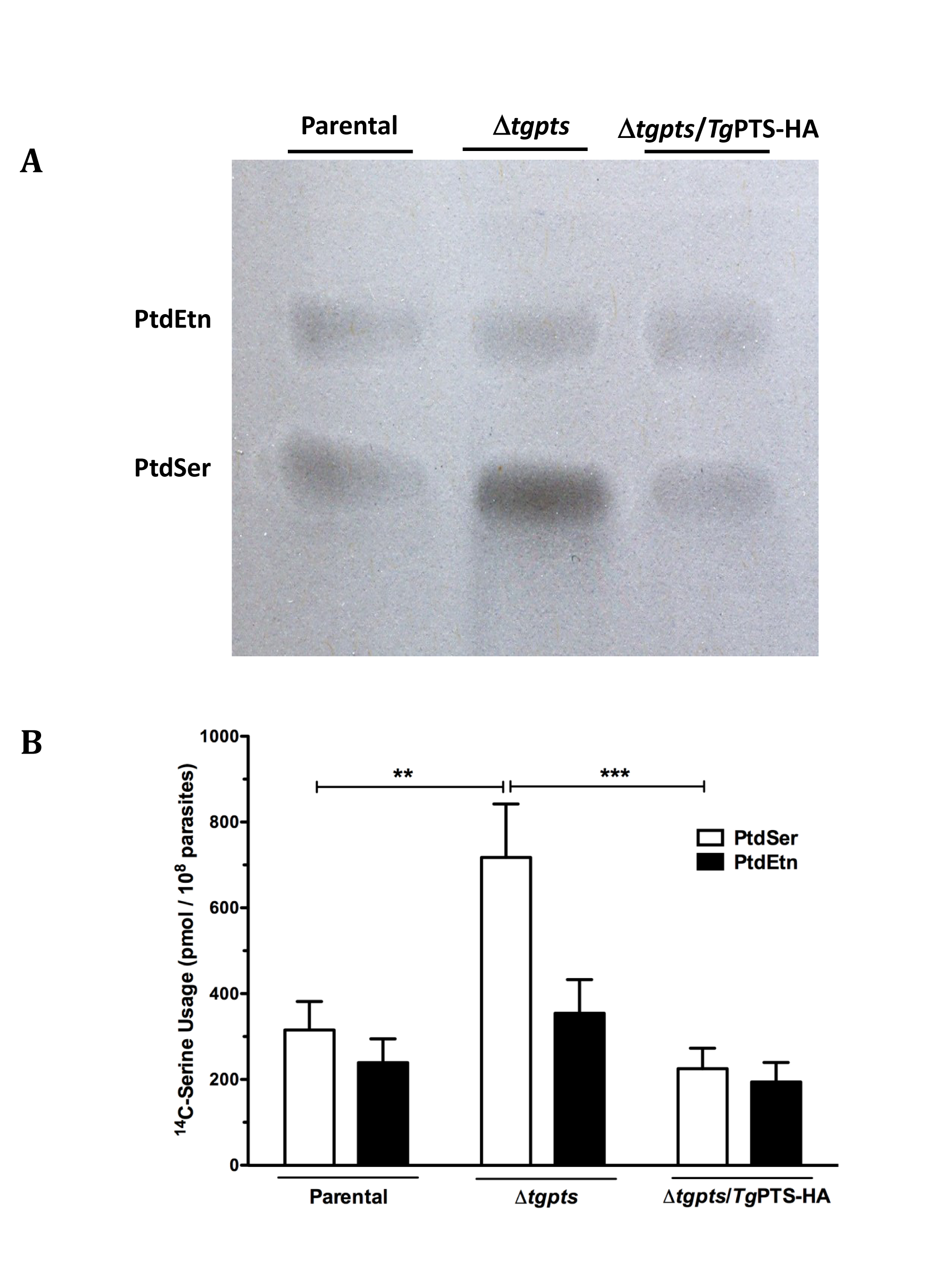

Supplement: S8 Fig — (A) Autoradiography of TLC-resolved parasite lipids following metabolic labeling with radioactive serine. Fresh extracellular parasites of the indicated strains were labeled with 14C-serine (2 μCi, 100 μM, 2 hr, 37°C, 5 x 10^7^ parasites). Solvent system used for TLC was chloroform/ethanol/water/triethylamine (30:35:7:35). Lipid bands were identified by migration with authentic standards. (B) Radiolabeled lipid bands from panel A were scraped for scintillation counting to determine the usage of serine into PtdSer and PtdEtn (mean ± SEM; n = 5 assays; **, p < 0.01; ***, p < 0.001). (TIFF) [file pbio.1002288.s009.TIFF]

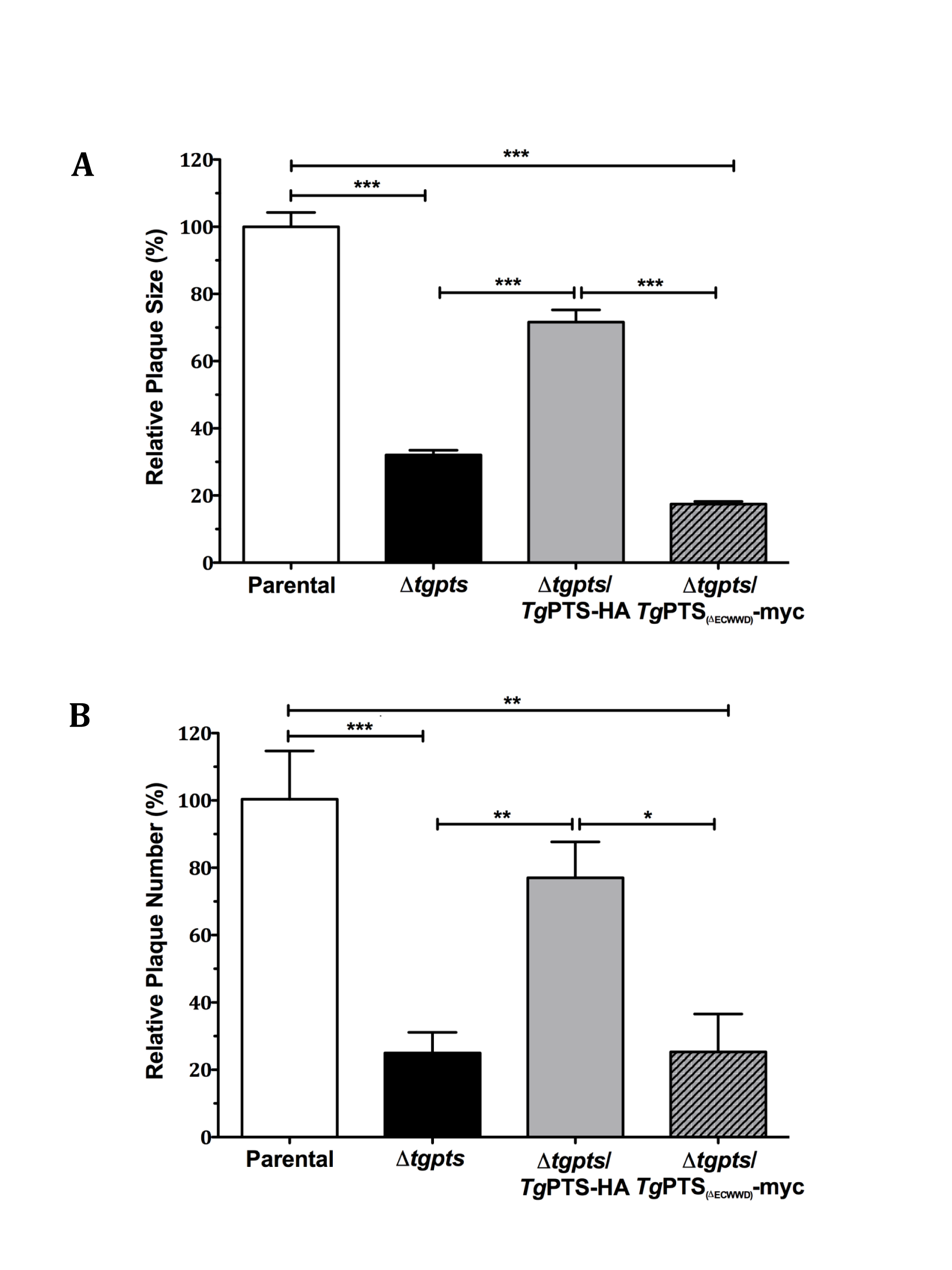

Supplement: S9 Fig — (A–B) Growth of the indicated parasite strains, as deduced by plaque assays. The decreased size (A) and number (B) of plaques formed by the Δtgpts mutant were significantly recovered by expression of a functional (wild-type) TgPTS-HA, but not by a catalytically-dead (TgPTS(ΔECWWD)-myc) isoform. In total, 50–130 plaques of each strain from 4 assays were analyzed (mean ± SEM; *p < 0.05, **p < 0.01, ***p < 0.001). (TIFF) [file pbio.1002288.s010.TIFF]

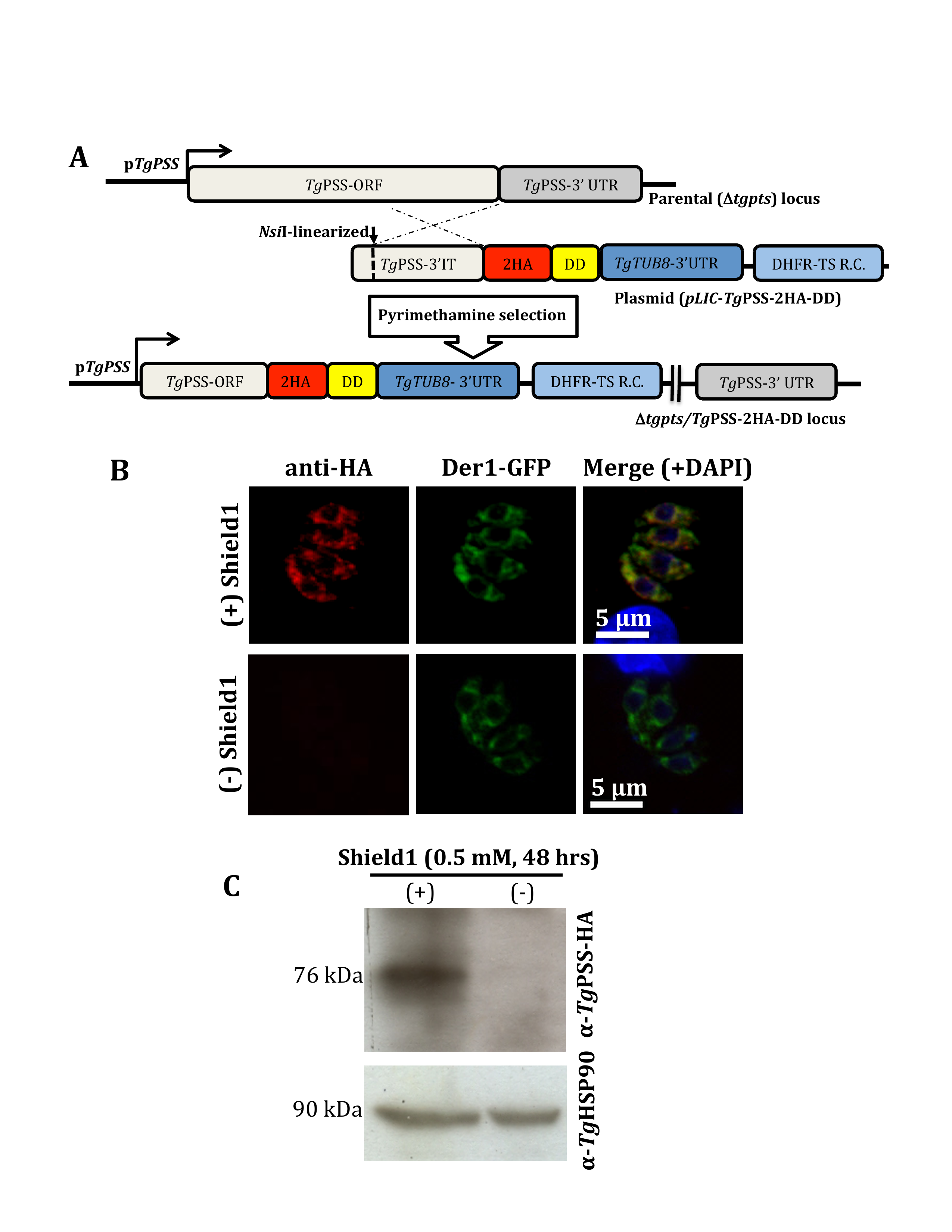

Supplement: S10 Fig — (A) Scheme showing the 3’-tagging of the TgPSS gene with the 2HA-DD epitope in the Δtgpts strain. Prior to transfecting parasites, the construct was linearized at the NsiI site to enable single homologous recombination at the 3’-end of the gene without perturbing the promoter sequence. Stable transgenic parasites (Δtgpts/TgPSS-2HA-DD) were generated by pyrimethamine selection. (B) Immunofluorescence images illustrating staining of TgPSS-2HA-DD with TgDer1-GFP (ER marker), and its regulation in the Δtgpts/TgPSS-2HA-DD strain. Parasites were cultured in Shield1 (0.5 μM, 24 hrs) prior to immunostaining. (C) Conditional regulation of TgPSS-2HA-DD by Shield1, as confirmed by immunoblot analyses. Parasitized cells were cultured in 0.5 μM Shield1 for 48 hr prior to detection with anti-HA antibody (TgHsp90, loading control). As expected, the anti-HA signal is absent in the untreated control samples in panels B and C. The absence of red staining in panel B (without Shield1) also precludes any “bleeding effect” from green to red channel. (TIFF) [file pbio.1002288.s011.TIFF]

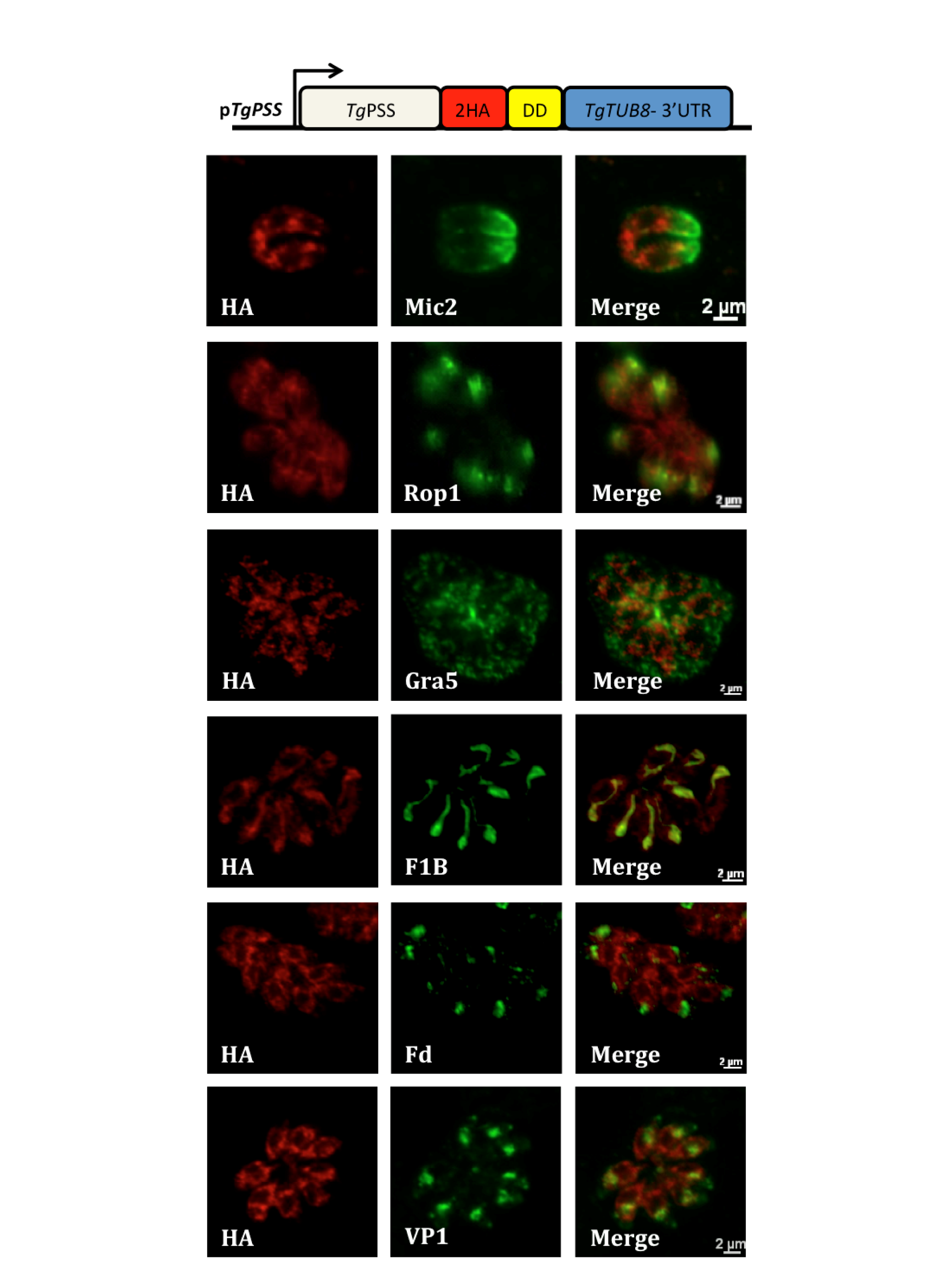

Supplement: S11 Fig — Stable transgenic parasites expressing TgPSS-2HA-DD under the control of its own promoter and TgTUB8-3’UTR were generated by 3’-insertional tagging of the gene, as described in S10 Fig. Cultures were treated with 0.5 μM Shield1 for 24 hr prior to immunostaining to visualize the fusion protein. Staining of Mic2, Rop1, Gra5, F1B, Fd, and VP1 proteins represents micronemes, rhoptries, dense granules, mitochondrion, apicoplast and acidocalcisomes/plant-like vacuole, respectively. Samples stained with anti-Rop1 and anti-Fd antibodies exhibited diffused and high background fluorescence, occasionally transecting with anti-HA. Most of the HA signal in the merged image however did not colocalize with any organelles except for mitochondrion and acidocalcisomes/plant-like vacuole, often superimposing ER extensions. (TIFF) [file pbio.1002288.s012.TIFF]

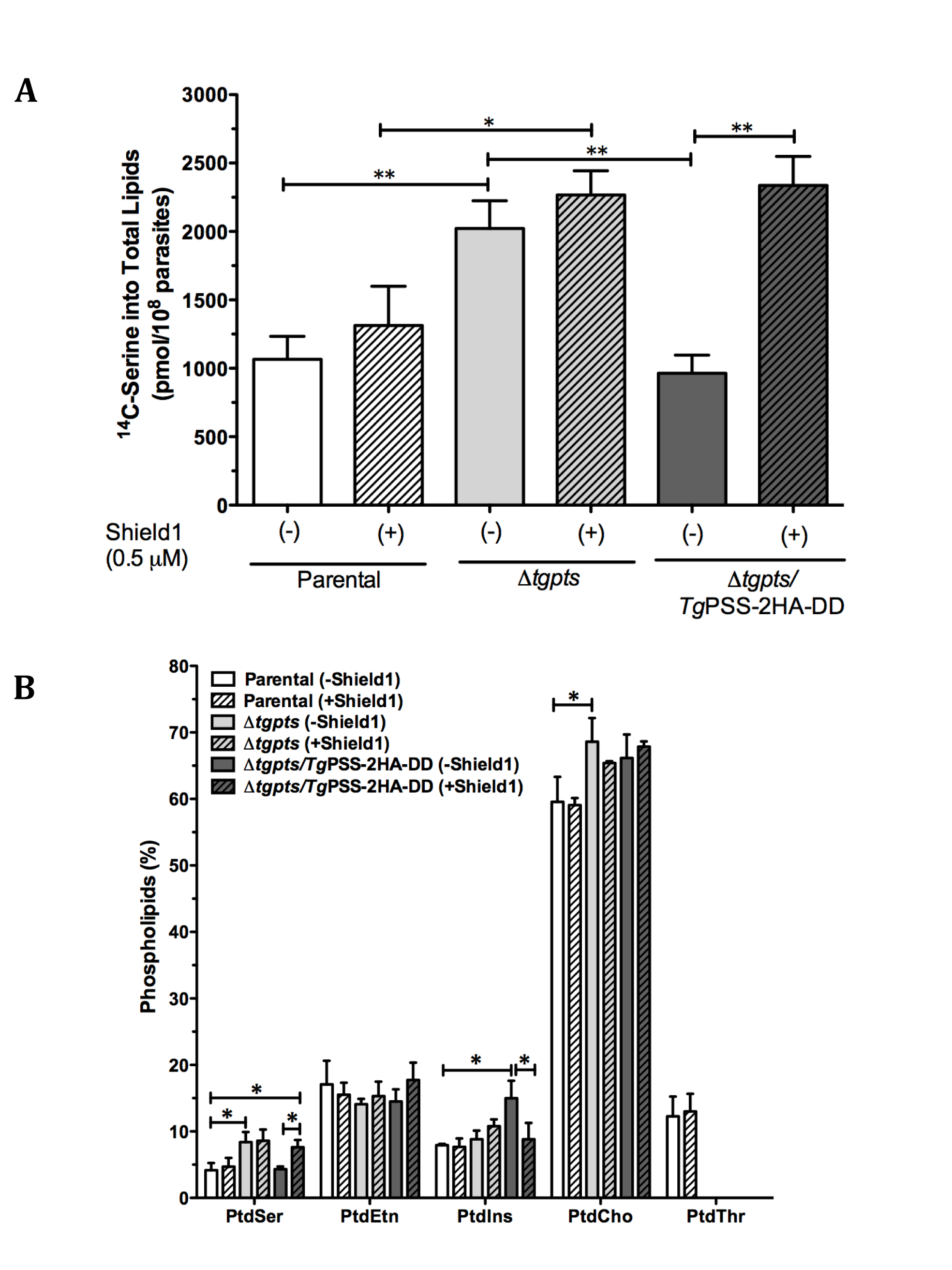

Supplement: S12 Fig — (A) Incorporation of 14C-serine into total lipid fraction of host-free parasites precultured during the intracellular phase without or with Shield1 (0.5 μM, 24 hrs). Labeling of parasites was done, as described in Fig 7A (mean ± SEM, n = 4 assays; *p < 0.05, **p < 0.01). (B) Quantification of lipid-phosphorus in the indicated parasites strains. Lipids (0.8–1 x 10^8^ tachyzoites) were resolved by two-dimensional TLC and subjected to lipid-phosphorus assay (mean ± SEM of 3 assays; *p < 0.05). The data in panels A–B also confirm the catalytic function of TgPSS in T. gondii. (TIFF) [file pbio.1002288.s013.TIFF]

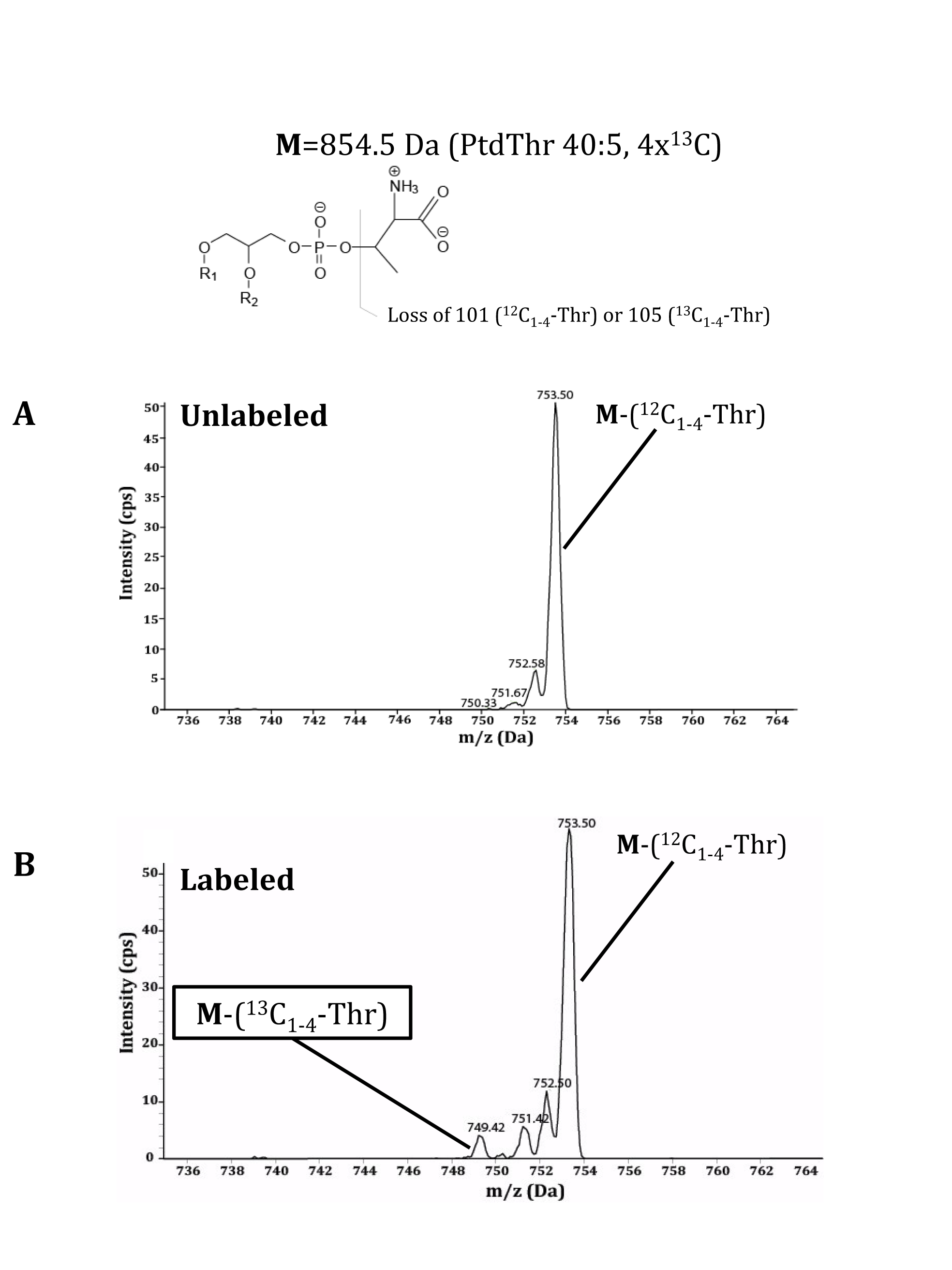

Supplement: S13 Fig — The parental parasites (RHΔku80-hxgprt -) were grown in HFF monolayers supplied with 0.4 mM 13C-threonine for 2 d. Lipids from syringe-released purified parasites were subjected to MS/MS analyses. Unlabeled samples were also analyzed to illustrate the natural abundance of 13C. The transitions 854.5–753.5 and 854.5–749.5 represent the neutral losses of 12C4-Thr (nl101) and 13C4-Thr (nl105), respectively, in the PtdThr peak (m/z 854.5, 40:5, 4x13C). 13C1-4-Thr indicates that all carbons are labeled in the threonine moiety (peak 749.5) of samples incubated with the stable isotope but not in the control, where the natural abundance of 4x labeled threonine is basically zero (no peak at 749.5 in unlabeled sample). (TIFF) [file pbio.1002288.s014.TIFF]
