# Supplementary material for: Phosphatidylthreonine and Lipid-Mediated Control of Parasite Virulence
Source: PLoS Biol. 2015 Nov 13;13(11):e1002288. doi: 10.1371/journal.pbio.1002288 (PMC4643901; doi:10.1371/journal.pbio.1002288)
Supplement: S1 Table — (PDF) [file pbio.1002288.s015.pdf]

Table S1: Oligonucleotides used in this study

Primer Name (restriction site)	Nucleotide Sequence (restriction site underlined)	Cloning Vector (research objective)
**Annotation of *Tg*PTS and *Tg*PSS**		
*Tg*PTS-F	ATGCAACTCCCTTCAAGA	*p*Drive (T/A-cloning for testing and sequencing *Tg*PTS (TGGT1_273540)
*Tg*PTS-R	TCACTGACTTCGTTCCATTTTCACG	
*Tg*PSS-F	ATGTGTCGGGGACCGCCGCT	*p*Drive (T/A-cloning for testing and sequencing *Tg*PSS (TGGT1_261480)
*Tg*PSS-R	TCACTCGTCTTTTTGGCCTTC	
**Expression and localization of *Tg*PTS and *Tg*PSS in *T. gondii* (RHΔ*ku80-hxgprt*$^-$ strain)**		
*Tg*PTS-F (*Nsi*I)	CTCATCATGCATATGCAACTCCCTTCAAGAAAGG	*pTgGRA1-UPKO* (Ectopic expression of *Tg*PTS-HA at the *TgUPRT* locus)
*Tg*PTS-HA-R (*Pac*I)	CTCATCTTAATTAATCAAGCGTAATCTGGAACATCGTA TGGGTACTGACTTCGTTCGATT	
*Tg*PSS-F (*EcoR*V)	CTCGATATCATGTGTCGGGGACCGCCGCT	*pTgSAG1-UPKO* (Ectopic expression of *Tg*PSS-HA at the *TgUPRT* locus)
*Tg*PSS-HA-R (*Pac*I)	CTCTTAATTAATCAAGCGTAATCTGGAACATCGTATGG GTACTCGTCTTTTTGGCCCTTCC	
**Making of Δ*tgpts* mutant in *T. gondii* (RHΔ*ku80-hxgprt*$^-$ strain)**		
*Tg*PTS-5'COS-F (*Not*I)	CTCATCGCGGCCGCGTTCGCCTCGAGTGCTTG	*pTKO-HXGPRT* (Cloning of the *Tg*PTS 5'COS)
*Tg*PTS-5'COS-R (*EcoR*I)	CTCATCGAATTCACGAGCCAGTGGAACGAC	
*Tg*PTS-3'COS-F (*Hpa*I)	CTCATCGTTAACAGCATCTTTATCGATGCGCT	*pTKO-HXGPRT* (Cloning of the *Tg*PTS 3'COS)
*Tg*PTS-3'COS-R (*Hpa*I)	CTCATCGTTAACTCACTGACTTCGTTCGATTTTC	
**Screening for 5' and 3' recombination in *T. gondii* (Δ*tgpts* strain)**		
*Tg*PTS-5'Scr-F	CGATTCCTTGAGAGCAACTG	*pDrive* (T/A-cloning of 5' PCR product for sequencing)
*Tg*PTS-5'Scr-R	GACGCAGATGTGCGTGTATC	
*Tg*PTS-3'Scr-F	ACTGCCGTGTGGTAAAATGAA	*pDrive* (T/A-cloning of 3' PCR product for sequencing)
*Tg*PTS-3'Scr-R	GCCATAGAGTTCATTGCGGACTC	
**Genetic complementation in *T. gondii* (Δ*tgpts* strain)**		
*Tg*PTS-F (*Nsi*I)	CTCATCATGCATATGCAACTCCCTTCAAGAAAGG	*pTgGRA1-UPKO* (Ectopic expression of *Tg*PTS-HA at the *TgUPRT* locus)
*Tg*PTS-HA-R (*Pac*I)	CTCATCTTAATTAATCAAGCGTAATCTGGAACATCGTA TGGGTACTGACTTCGTTCGATTTTCACG	
*Tg*PTS$_{(\Delta ECWWD)}$-P1-F (*Nsi*I)	CTCATCATGCATATGCAACTCCCTTCAAGAAAGG	*pTgGRA1-UPKO* (2-step cloning for ectopic expression of *Tg*PTS$_{(\Delta ECWWD)}$-myc at the *TgUPRT* locus)
*Tg*PTS$_{(\Delta ECWWD)}$-P1-R (*Sbf*I)	CTCATCCCTGCAGGGCGCAGAGTTCGGGGACGAG	
*Tg*PTS$_{(\Delta ECWWD)}$-P2-F (*Nsi*I)	CTCATCATGCATAGCATCTTTATCGATGCGCTG	
*Tg*PTS$_{(\Delta ECWWD)}$-P2-myc-R (*Pac*I)	CTCATCTTAATTAATCACAGATCTTCTTCAGAAATAAG TTTTTGTTCCTGACTTCGTTCGATTTTCACGT	
**3'-tagging of *Tg*PSS gene locus with 2HA-DD tag (Δ*tgpts* strain)**		
*Tg*PSS-3'IT-2HA-DD-F	TACTTCCAATCCAATTTAATGCGACGGGGAAGTCCTTT GG	*pLIC-2HA-DD-DHFR* (Ligation-independent cloning for expression of *Tg*PSS-2HA-DD fusion protein)
*Tg*PSS-3'IT-2HA-DD-R	TCCTCCACTTCCAATTTTAGCCTCGTCTTTTTGGCCTTC C	
**Expression of genes flanking the *TgPTS* locus in *T. gondii* (RHΔ*ku80-hxgprt*$^-$, Δ*tgpts* and Δ*tgpts*/*Tg*PTS-HA strains)**		
TGGT1_273550-F	ATGCATTGTCAACTAGGAGGC	ORF-specific PCR of TGGT1_273550
TGGT1_273550-R	TTACAGTGTCGAACTGGGGTC	

TGGT1_273530-F	ATGTTGAAGACACCAGTAACGGT	ORF-specific PCR of TGGT1_273530
TGGT1_273530-R	TCAAGCGACAGATAGGTCGTC	
**Quantification of *T. gondii* infection in mouse brain tissue**		
*Tg*B1-F	TCCCCTCTGCTGGCGAAAAGT	Quantification of parasite cyst burden by qPCR method
*Tg*B1-R	AGCGTTCGTGGTCAACTATCGATTG	
*Mm*ASL-F	TCTTCGTTAGCTGGCAACTCACCT	Quantification of a reference gene (mouse host) for normalization of parasite cyst burden by qPCR method
*Mm*ASL-R	ATGACCCAGCAGCTAAGCAGATCA	
**Expression of *Tg*PTS and *Tg*PSS in *E. coli* (M15/pREP4 strain)**		
*Tg*PTS-F (*Bgl*II)	CTCATCAGATCTATGCAACTCCCTTCAAGAAAGG	*pQE60* (expression of *Tg*PTS-6xHis)
*Tg*PTS-His-R (*Bgl*II)	CTCATCAGATCTCTGACTTCGTTCGATTTTCACG	
*Tg*PSS-F (*Bgl*II)	CTCATCAGATCTATGTCGGGGACTGCCGCT	*pQE60* (expression of *Tg*PSS-6xHis)
*Tg*PSS-His-R (*Bgl*II)	CTCATCAGATCTCTCGTCTTTTTGGCCTTCCAACA	
